# Dynamic interplay between sortilin and syndecan-1 contributes to prostate cancer progression

**DOI:** 10.1038/s41598-023-40347-7

**Published:** 2023-08-18

**Authors:** Joanna Lazniewska, Ka Lok Li, Ian R. D. Johnson, Alexandra Sorvina, Jessica M. Logan, Carmela Martini, Courtney Moore, Ben S.-Y. Ung, Litsa Karageorgos, Shane M. Hickey, Sarita Prabhakaran, Jessica K. Heatlie, Robert D. Brooks, Chelsea Huzzell, Nicholas I. Warnock, Mark P. Ward, Bashir Mohammed, Prerna Tewari, Cara Martin, Sharon O’Toole, Laura Bogue Edgerton, Mark Bates, Paul Moretti, Stuart M. Pitson, Stavros Selemidis, Lisa M. Butler, John J. O’Leary, Douglas A. Brooks

**Affiliations:** 1 Clinical and Health Sciences, University of South Australia, Adelaide, SA 5000 Australia; 2 Department of Anatomical Pathology, College of Medicine and Public Health, Flinders University, Bedford Park, SA 5042 Australia; 3 Centre for Cancer Biology, University of South Australia and SA Pathology, Adelaide, SA 5000 Australia; 4 Department of Histopathology, Trinity College Dublin, Dublin 8, Ireland; 5 School of Health and Biomedical Sciences, STEM College, RMIT University, Bundoora, VIC 3083 Australia; 6 South Australian ImmunoGENomics Cancer Institute and Freemasons Centre for Male Health and Wellbeing, University of Adelaide, Adelaide, SA 5000 Australia; 7 Solid Tumour Program, Precision Cancer Medicine Theme, South Australian Health and Medical Research Institute, Adelaide, SA 5000 Australia

**Keywords:** Cancer metabolism, Prostate cancer, Tumour biomarkers, Endosomes

## Abstract

Prostate cancer (PCa) development and progression relies on the programming of glucose and lipid metabolism, and this involves alterations in androgen receptor expression and signalling. Defining the molecular mechanism that underpins this metabolic programming will have direct significance for patients with PCa who have a poor prognosis. Here we show that there is a dynamic balance between sortilin and syndecan-1, that reports on different metabolic phenotypes. Using tissue microarrays, we demonstrated by immunohistochemistry that sortilin was highly expressed in low-grade cancer, while syndecan-1 was upregulated in high-grade disease. Mechanistic studies in prostate cell lines revealed that in androgen-sensitive LNCaP cells, sortilin enhanced glucose metabolism by regulating GLUT1 and GLUT4, while binding progranulin and lipoprotein lipase (LPL) to limit lipid metabolism. In contrast, in androgen-insensitive PC3 cells, syndecan-1 was upregulated, interacted with LPL and colocalised with β_3_ integrin to promote lipid metabolism. In addition, androgen-deprived LNCaP cells had decreased expression of sortilin and reduced glucose-metabolism, but increased syndecan-1 expression, facilitating interactions with LPL and possibly β_3_ integrin. We report a hitherto unappreciated molecular mechanism for PCa, which may have significance for disease progression and how androgen-deprivation therapy might promote castration-resistant PCa.

## Introduction

Prostate cancer (PCa) is the second most common cancer diagnosed in men worldwide and the fifth leading cause of cancer death^1^. PCa has a complex biology and is subject to genetic diversity, morphological heterogeneity and clinical variability^2^. Androgen signalling promotes primary tumour development, however, over time the androgen receptor gene undergoes amplification and mutation, which drives cancer progression even when androgen levels are low. Moreover, androgen-deprivation therapy (ADT), applied in the early stages of the disease is only effective for a limited amount of time, and the disease inevitably progresses to a castration-resistant form with poor survival rates (30% at 5 years)^3^. Currently, we do not have a suitable explanation as to why the biology of PCa limits effective treatment intervention.

Glucose and lipid metabolic reprogramming are key features in PCa establishment and progression, which underpins the development of resistance to therapeutic interventions, including ADT^4^. In early stages of the disease, metabolic modifications are controlled by androgen signalling and indeed, androgens induce glycolysis, oxidative phosphorylation and fatty acid oxidation^5^. Although alterations in lipid metabolism occur early in tumour development, the fully-established lipogenic phenotype is commonly associated with castration-resistant cancer^6,7^. The capacity to utilise alternative energy sources at different stages of cancer progression is crucial for responding to variable environmental factors, such as nutrient and oxygen availability; which necessitates a heterogenous and adaptable metabolic profile^6,8^. The cellular machinery involved in the metabolic programming of PCa remains unclear.

Endosomes and lysosomes have a critical role in cellular uptake, intracellular trafficking/cargo sorting, signalling, inflammation, and metabolism, which are all hallmarks of PCa pathogenesis^9^. We have previously reported altered regulation of the endosome-lysosome system in PCa^10,11^ and more recently demonstrated that sortilin and syndecan-1 delineate PCa pathogenesis in patient tissues^12^. Sortilin and syndecan-1 have been implicated in the development and progression of many malignancies, including PCa^13,14^. Sortilin (a critical Golgi and endosome sorting receptor) and syndecan-1 (a cell surface and endosomal proteoglycan) have a range of binding partners that are implicated in the control of glucose and lipid metabolism, which may be central to PCa metabolic programming^15–17^.

The differential detection of sortilin and syndecan-1 in well- and poorly-formed malignant glands aligns respectively with low- and high-grades of PCa^12,18^, suggesting that these proteins are central to PCa pathogenesis and disease progression. We hypothesised that the high expression of sortilin in low-grade PCa aligns with the regulation of glucose metabolism in LNCaP cells, while elevated syndecan-1 expression in high-grade PCa aligns with lipid metabolism in PC3 cells. Our study alludes to a mechanism of PCa progression that involves the dynamic balance between sortilin and syndecan-1, which aligns respectively with glucose or lipid metabolism.

## Results

### Sortilin and syndecan-1 protein expression in patient tissue samples is recapitulated in prostate cell line models

**Figure 1:**
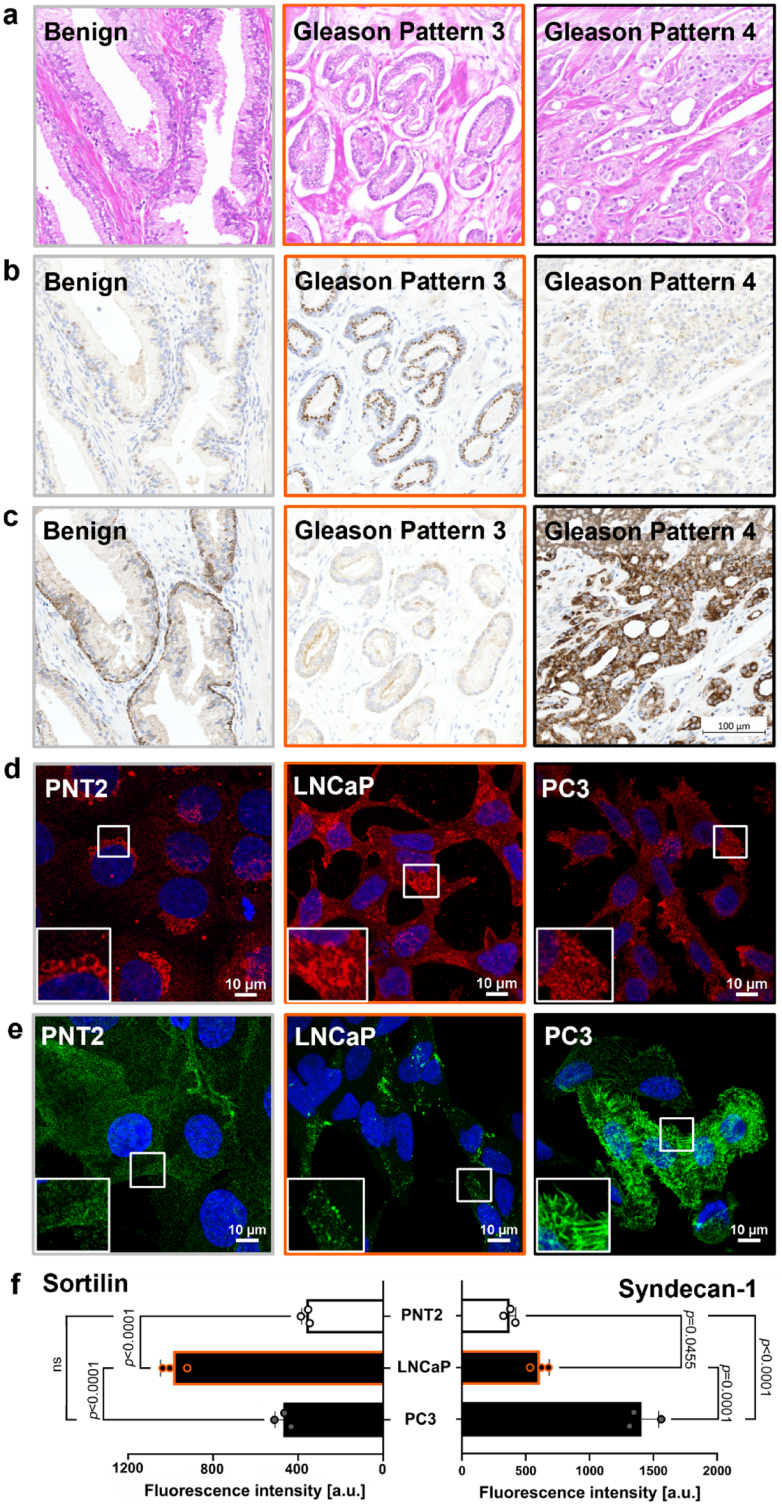
Sortilin and syndecan-1 immunolabelling depicts pathogenesis of PCa, which aligns with immunofluorescence in prostate cell line models. (**a**) Representative serial tissue sections stained with hematoxylin and eosin (H&E), showing benign glands, Gleason pattern 3 and Gleason pattern 4. (**b**) Benign glands, Gleason pattern 3 and Gleason pattern 4 were IHC-labelled for sortilin and (**c**) for syndecan-1. (**d**) Representative maxIP confocal images, showing immunolabelling of prostate cell lines with anti-sortilin antibodies and (**e**) anti-syndecan-1 antibodies. Scale bars; 10 µm. (**f**) Quantification of sortilin and syndecan-1 fluorescence intensity in prostate cell lines. Data are presented as mean ± SD, n = 3 (independent experiments), one-way ANOVA test. Scale bar for tissue images (**a–c**)is depicted in (**c**); 100 µm.

PCa tissue with well-formed glands (Gleason Pattern 3) (Fig. 1a) exhibited characteristic sortilin immunolabelling, with a perinuclear granular distribution (Fig. 1b), while syndecan-1 displayed minimal immunolabelling (Fig. 1c). In contrast, poorly-formed glands (Gleason pattern 4) (Fig. 1a) had intense syndecan-1 (Fig. 1c), but minimal sortilin immunolabelling (Fig. 1b). These immunolabelling patterns were representative of a large cohort of tissue samples from PCa patients (n > 100), which were previously used to validate these markers where there was a significant change in sortilin and syndecan-1 expression and distribution that enabled more reliable IHC-assisted grading and better stratification of patients^12,18^.

Approximately 90% of sortilin is known to localise to the Golgi apparatus where it facilitates endosome trafficking, while the remainder is detected in endosome compartments and is known to recycle at the cell surface^19^. Immunofluorescence demonstrated a differential distribution for sortilin between the non-malignant and malignant prostate cell lines (Fig. 1d). In non-malignant PNT2 cells, sortilin was detected primarily in the perinuclear region, consistent with the Golgi apparatus, while in malignant LNCaP cells there was also sortilin labelling in vesicular compartments at, or close to the cell surface. In malignant PC3 cells, sortilin displayed a dispersed immunolabelling pattern of vesicular compartments, with some protein located at or near the cell surface (Fig. 1d). Analysis of the fluorescence intensity showed ~ twofold higher amount of sortilin in LNCaP cells than in PC3 and PNT2 cells (Fig. 1f).

In PNT2 cells, syndecan-1 was dispersed, with low-intensity immunolabelling in vesicular compartments throughout the cytoplasm and was also present at or near the plasma membrane (Fig. 1e). In LNCaP cells, the intensity of syndecan-1 labelling was significantly higher than in PNT2 cells (Fig. 1e) and evident in punctate vesicular compartments as well as at, or in proximity to, the plasma membrane (Fig. 1e). In contrast, PC3 cells showed a distinctive labelling pattern, with the majority of syndecan-1 localised at or near the cell membrane. Quantification of syndecan-1 fluorescence intensity (Fig. 1f) confirmed higher expression of this protein in malignant cell lines as compared to PNT2 cells, with the highest intensity in PC3 cells.

While some aspects of the immunolabelling of sortilin and syndecan-1 in LNCaP cells were consistent with that observed in well-formed glands, and PC3 cells were mostly representative of the immunohistochemistry (IHC) in poorly-formed glands (Fig. 1), the LNCaP and PC3 cell lines have limitations as models of early and late stage disease as they were both derived from patients with metastatic disease (there is no commercially available cell line model of primary prostatic adenocarcinoma^20^). The cell lines therefore displayed some features of sortilin and syndecan-1 expression, which enabled them to be used for the proposed mechanistic studies.

### Sortilin controls glucose uptake in PCa cells and glucose regulates expression of sugar-metabolism-related proteins

**Figure 2:**
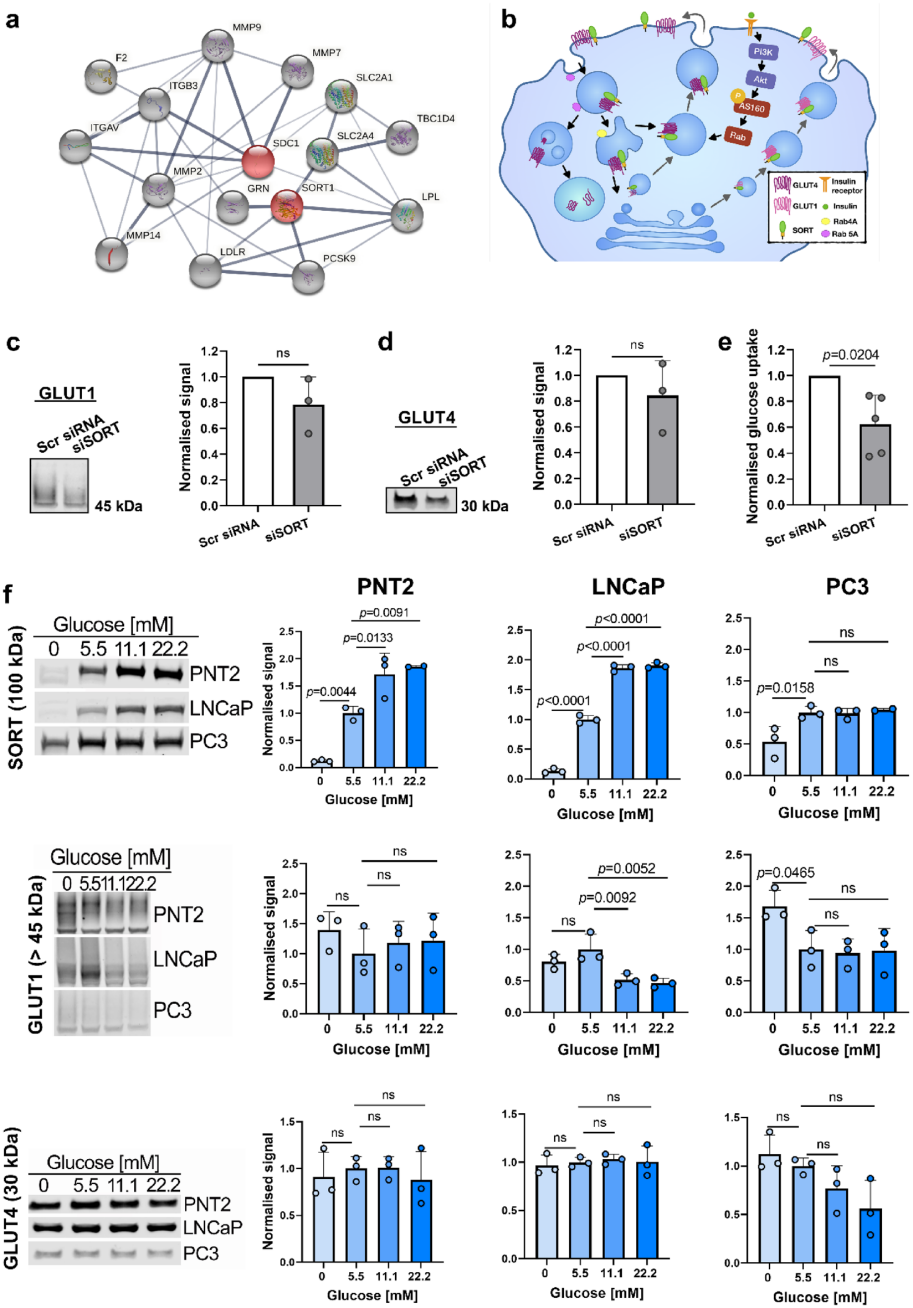
Sortilin controls glucose uptake in PCa cells and glucose alters expression of sugar metabolism-related proteins. (**a**) Sortilin (SORT1) and syndecan-1 (SDC1)-interacting proteins, which are implicated in PCa development and progression. Red nodes; SORT and SDC1, gray nodes; proteins crucial for PCa metabolism relevant to this study. Line thickness indicates the strength of data support. (**b**) diagram showing involvement of sortilin (SORT) in GLUT4 and potentially GLUT1 pathways. (**c**) Expression of GLUT1, (**d**) GLUT4 and (**e**) glucose uptake in LNCaP cells after sortilin knockdown (siSORT) as compared to scrambled siRNA (Scr siRNA). (**f**) Expression of SORT, GLUT1 and GLUT4 in different glucose concentrations in prostate cell lines. Western blotting signal was normalised using total protein staining. Data are presented as mean ± SD, in (**c,d**,**f**) n = 3 (independent experiments), in (**e**) n = 5 (independent experiments), in (**c–e**), one-sample t-test, in (**f**) one-way ANOVA test, where samples were normalized and compared to 5.5 mM glucose.

Using STRING database, we identified a network of potential interactions^21^ for sortilin and syndecan-1 (Fig. 2a), which may be involved in PCa pathogenesis and analysed the expression of these genes in LNCaP and PC3 cells using RNA sequencing (RNAseq) (Supplementary Fig. S1 and Supplementary Table S1). Furthermore, we depicted an interaction of sortilin with GLUT4^22^ and GLUT1 (Fig. 2b). siRNA against sortilin (siSORT) reduced its expression by 96% (Supplementary Fig. S2). siSORT did not have a significant impact on GLUT1 (Fig. 2c) and GLUT4 (Fig. 2d) expression. However, sortilin knock-down significantly lowered glucose uptake, as compared to scrambled-siRNA-treated LNCaP cells (Fig. 2e).

Next, we investigated if glucose concentration (0–22.2 mM) influences the expression of sortilin, GLUT1 and GLUT4. A concentration of 5.5 mM glucose was used as a reference level, as this is within normal physiological range (3.9–5.5 mM in blood)^23^. Western blotting demonstrated that glucose deprivation (0 mM) for 48 h caused a reduction of sortilin in all cell lines, while high glucose concentrations (11.1 and 22.2 mM) led to a significant increase in sortilin in PNT2 and LNCaP cells (Fig. 2f). The amount of GLUT1 remained unchanged in response to different glucose concentrations in PNT2 cells, while in PC3 cells the absence of glucose resulted in ~ 1.7-fold increase in GLUT1. The amount of GLUT1 in LNCaP cells was comparable at 0 and 5.5 mM glucose, but dropped ~ twofold at 11.1 and 22.2 mM (Fig. 2f). There was no statistically significant impact on GLUT4 expression in any cell line (Fig. 2f).

### Sortilin likely regulates GLUT1 and GLUT4 via AS160/Rab10/Rab14 pathway

**Figure 3:**
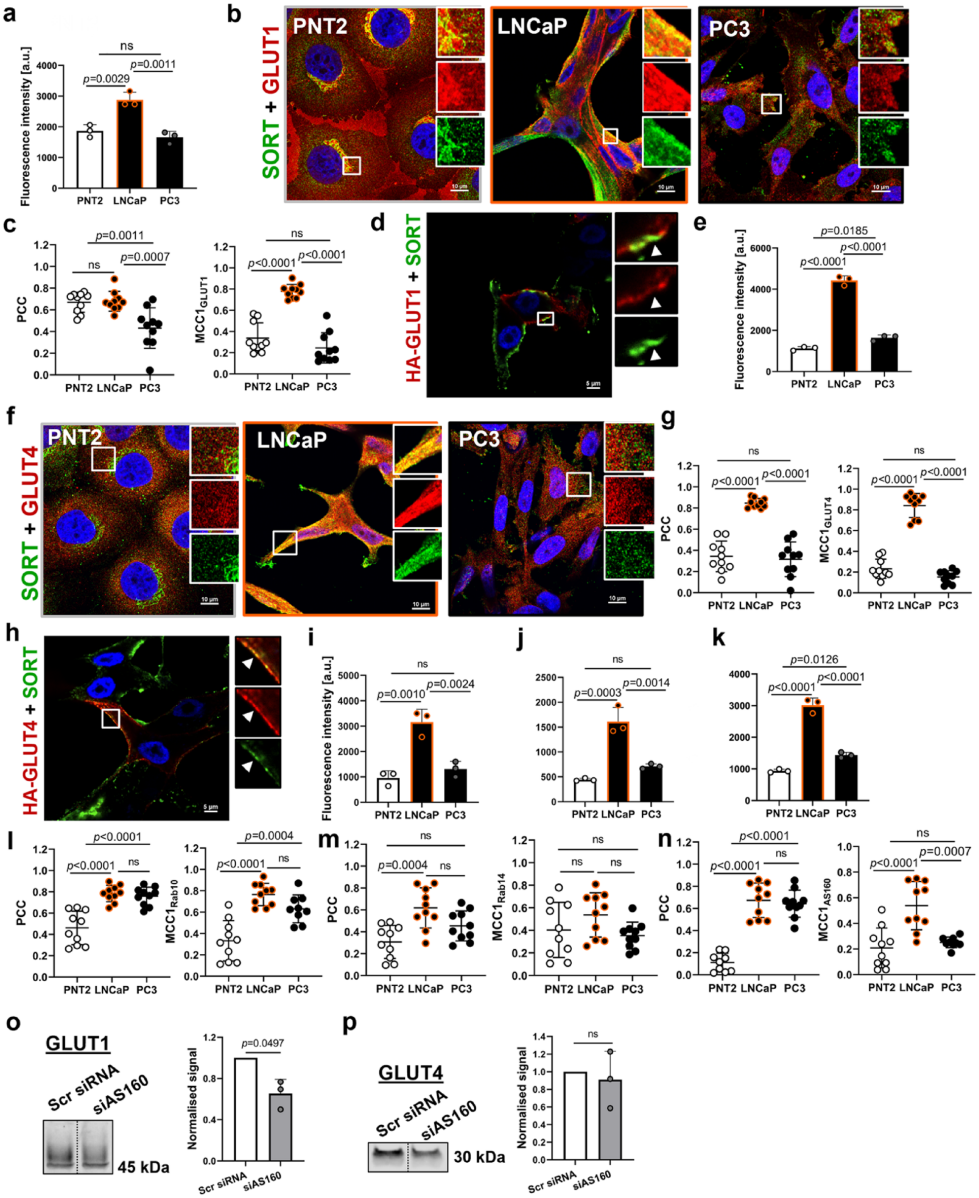
Sortilin (SORT) likely regulates GLUT1 and GLUT4 via AS160/Rab10/Rab14 pathway. (**a**) Quantification of GLUT1 fluorescence intensity. (**b**) Representative confocal images showing co-labelling of cells with anti-GLUT1 (red) and anti-SORT (green) antibodies. Scale bars; 10 µm. (**c**) Quantification of colocalisation between GLUT1 and SORT. (**d**) Representative confocal image showing colocalisation between HA-GLUT1 (red) and SORT (green) at the cell membrane (arrowheads) of LNCaP cells. Scale bars; 5 µm. (**e**) Quantification of GLUT4 fluorescence intensity. (**f**) Representative confocal images showing co-labelling of cells with anti-GLUT4 (red) and anti-SORT (green) antibodies. Scale bars; 10 µm. (**g**) Quantification of colocalisation between GLUT4 and SORT. (**h**) Representative confocal image showing colocalisation between HA-GLUT4 (red) and SORT (green) at the cell membrane (arrowheads) of LNCaP cells. Scale bars; 5 µm. (**i**) Quantification of Rab10, (**j**) Rab14 and (**k**) AS160 fluorescence intensity. (**l**) Quantification of colocalisation between Rab10 and SORT, (**m**) Rab14 and SORT and (**n**) AS160 and SORT. (**o**) GLUT1 and (**p**) GLUT4 expression in LNCaP cells after AS160 knockdown (siAS160) as compared to scrambled siRNA (Scr siRNA). Western blotting bands from the same membrane were shifted towards each other. Western blotting signal was normalised using total protein staining. Data are presented as mean ± SD, in (**c**,**g**,**l,m**,**n**) n = 10 representative ROIs, in (**a**,**e**,**i,j**,**k**) n = 3 (independent experiments), one-way ANOVA, in (**o**,**p)** n = 3 (independent experiments), one-sample t-test.

Quantification of fluorescence intensity demonstrated significantly higher expression of GLUT1 in LNCaP cells, compared to PNT2 and PC3 cells (Fig. 3a). Analysis of GLUT1 distribution and its colocalisation with sortilin, indicated that in PNT2 cells both proteins had a strong perinuclear localisation (Fig. 3b). In LNCaP cells, GLUT1 was distributed to the cell membrane and intracellular compartments and largely colocalised with sortilin; while in PC3 cells this protein showed mainly diffuse vesicular localisation and little overlap with sortilin (Fig. 3b). Quantification of Pearson’s correlation coefficient (PCC) between sortilin and GLUT1 revealed a moderate colocalisation in PNT2 and LNCaP cells and a weaker colocalisation in PC3 cells. The amount of GLUT1 fluorescence overlapping with sortilin fluorescence (MCC1_GLUT1_) was significantly higher in LNCaP cells as compared to the two other cell lines (Fig. 3c). Endogenous sortilin also colocalised with HA-GLUT1 (HA-tag was attached to the extracellular loop of GLUT1) at the plasma membrane (Fig. 3d).

The fluorescence intensity of GLUT4 was ∽fourfold higher in LNCaP cells, as compared to PNT2 and PC3 cells (Fig. 3e). In PNT2 and PC3 cells, GLUT4 displayed intracellular distribution and limited colocalisation with sortilin (Fig. 3f,g). In LNCaP cells, GLUT4 was also observed at or near the plasma membrane, and there was a significant colocalisation with sortilin (Fig. 3f,g). Labelling of surface proteins revealed colocalisation between endogenous sortilin and HA-GLUT4 (HA-tag attached to the extracellular loop of GLUT4) at the plasma membrane of LNCaP cells (Fig. 3h). In addition, over-expression of sortilin-GFP with GLUT4-mCherry in live LNCaP cells showed colocalisation of these proteins both in intracellular vesicles and at the plasma membrane (Supplementary Fig. S3). Time-lapse imaging revealed that sortilin-GFP and GLUT4-mCherry vesicles trafficked between intracellular compartments from the trans-Golgi network to the plasma membrane (Supplementary Video S1).

In adipocytes and myocytes, phosphorylation of Akt substrate of 160 kDa (AS160) leads to activation of Rab14 and Rab10 GTPases, which mediate GLUT4 vesicle delivery to the plasma membrane via endosomal compartments, and facilitates their translocation and docking at the cell surface, respectively^24^ (Fig. 2b). The pattern of Rab10, Rab14 and AS160 expression matched that of GLUT4, where LNCaP cells demonstrated ∽threefold higher fluorescence intensity compared to PNT2 and PC3 cells (Fig. 3i–k). Furthermore, Rab10 showed a strong correlation and overlap with sortilin in LNCaP and PC3 cells, but not in PNT2 cells (Fig. 3l). While there was some colocalisation between Rab14 and sortilin in LNCaP compared to PNT2 cells (PCC), there was little overlap and no differences between cell lines (MCC1_Rab14_) (Fig. 3m). In contrast, AS160 and sortilin had significant PCC in LNCaP and PC3 cells, compared to PNT2 cells, and there was a moderate overlap in LNCaP cells, which was significantly different from PNT2 and PC3 cells (MCC1_AS160_, Fig. 3n). Knockdown of AS160 (siAS160) (Supplementary Fig. S4) led to a significant reduction in GLUT1 expression in LNCaP cells (Fig. 3o) but had less impact on GLUT4 (Fig. 3p). RNAseq results revealed that genes encoding for glucose transporters and their recycling machinery (*SLC2A4, SLC2A1, Rab11A, Rab4a*) were upregulated in LNCaP cells as compared to PC3 cells (Supplementary Fig. S1, Supplementary Table S1).

### Sortilin interacts with progranulin and LPL which are involved in lipid metabolism and cancer progression

**Figure 4:**
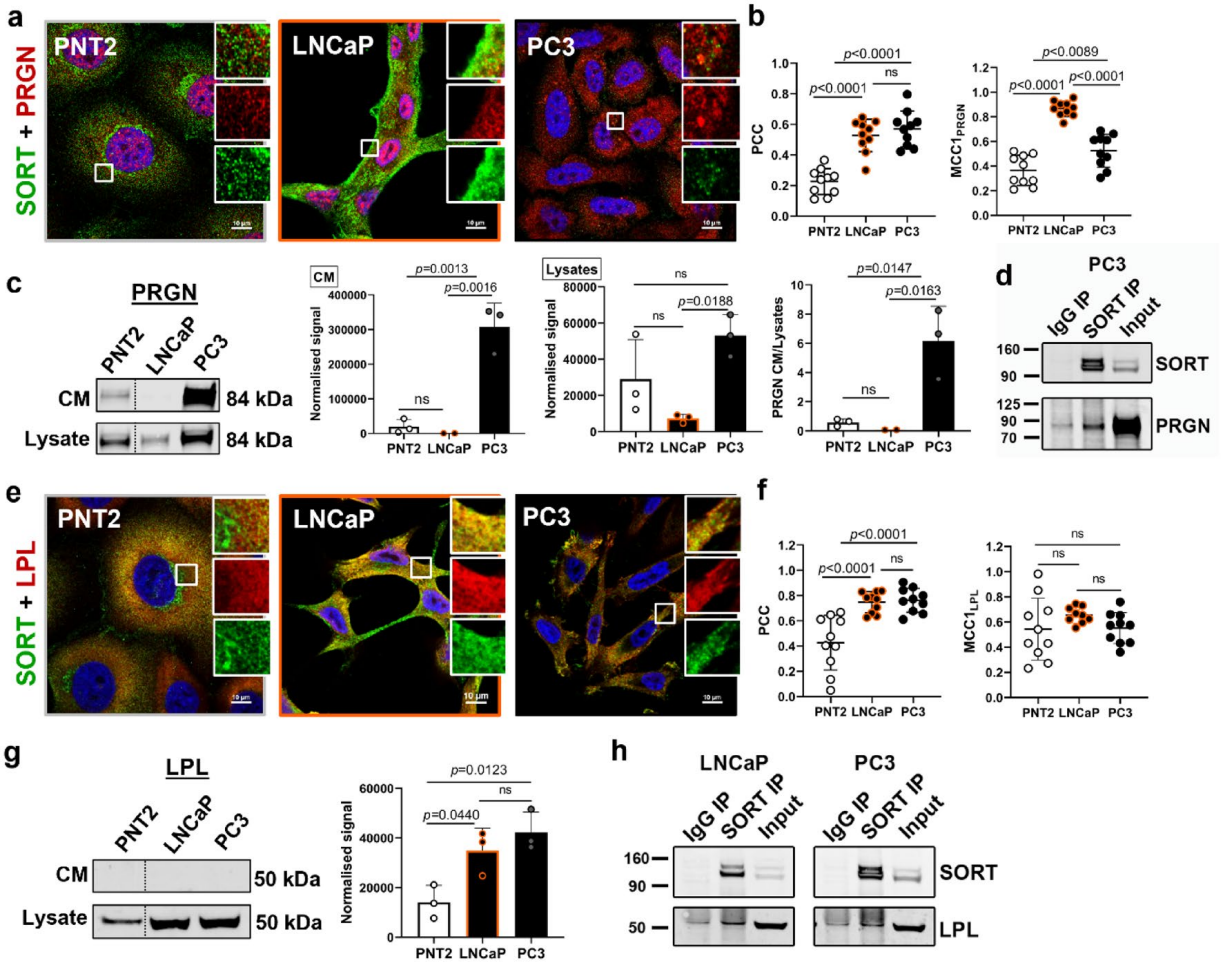
Sortilin (SORT) interacts with progranulin (PRGN) and LPL in PCa cells and PRGN is secreted by aggressive PC3 cells. (**a**) Representative confocal images showing co-labelling of cells with anti-PRGN (red) and anti-SORT (green) antibodies. Scale bars; 10 µm. (**b**) Quantification of colocalisation between PRGN and SORT. (**c**) Amount of PRGN in conditioned media (CM) and corresponding cell lysates with quantification of band densities and ratio of PRGN in CM to PRGN in cell lysates. (**d**) Endogenous SORT was immunoprecipitated (IP) with anti-SORT antibodies and PRGN was detected by Western blotting. Input; cell lysate. (**e**) Representative confocal images showing co-labelling of cells with anti-LPL (red) and anti-SORT (green) antibodies. Scale bars; 10 µm. (**f**) Quantification of colocalisation between LPL and SORT. (**g**) Amount of LPL in CM and corresponding cell lysates with quantification of band densities in cell lysates. In (**c**) and (**g**) PNT2 bands from the same membrane were shifted towards LNCaP and PC3 bands. Western blotting signal was normalised using total protein staining. (**h**) Endogenous SORT was immunoprecipitated (IP) with anti-SORT antibodies and LPL was detected by Western blotting. Input; cell lysate. Data are presented as mean ± SD, in (**b**,**f**) n = 10 representative ROIs, in (**c**,**g**) n = 3 (independent experiments), one-way ANOVA.

Sortilin regulates internalisation and degradation of secreted proteins, such as progranulin and LPL^16,25^, which have been implicated in PCa progression^26,27^. Progranulin was detected in all cell lines but was most evident in PC3 cells (Fig. 4a). Whilst PNT2 cells displayed minimal signal correlation (PCC) between sortilin and progranulin, as compared to PCa cells, the overlap (MCC1_PRGN_) was significantly higher in LNCaP than PC3 cells (Fig. 4a,b). Sortilin knockdown in LNCaP cells resulted in significant increase in progranulin as compared to scrambled siRNA-treated cells (Supplementary Fig. S5a). LNCaP cell extracts contained less progranulin, compared to PNT2 cells, and no detectable protein in the culture medium (Fig. 4c). In contrast, PC3 cells had high amounts of progranulin in both the cell extracts and culture medium, compared to PNT2 and LNCaP cells (Fig. 4c), and the ratio of secreted protein to intracellular progranulin was also the highest in PC3 cells (Fig. 4c). High expression of progranulin gene (*GRN*) in PC3 versus LNCaP cells was also detected by RNAseq (Supplementary Fig. S1 and Supplementary Table S1). The interaction between endogenous sortilin and progranulin was confirmed by co-immunoprecipitation assay in PC3 cells (Fig. 4d) but this association was not found in LNCaP cells (Supplementary Fig. S5b), possibly due to small amounts of progranulin in these cells.

There was a significant amount of colocalisation between sortilin and LPL, which was highest in LNCaP and PC3 cells (Fig. 4e,f). Knockdown of sortilin in LNCaP cells caused a decrease in LPL (Supplementary Fig. 5c). Western blotting showed higher amounts of LPL in LNCaP and PC3 cells, compared to PNT2 cells (Fig. 4g). There was no detectable LPL in the culture media of these prostate cell lines (Fig. 4g). Results of co-immunoprecipitation assay indicated that there was an interaction between endogenous sortilin and LPL in LNCaP and PC3 cells (Fig. 4h), but it should be noted that the co-immunoprecipitation with the IgG negative control still had detectable LPL, albeit less than the sortilin immunoprecipitation.

### Syndecan-1 interacts with LPL and colocalises with β_3_ integrin in androgen-insensitive cells

**Figure 5:**
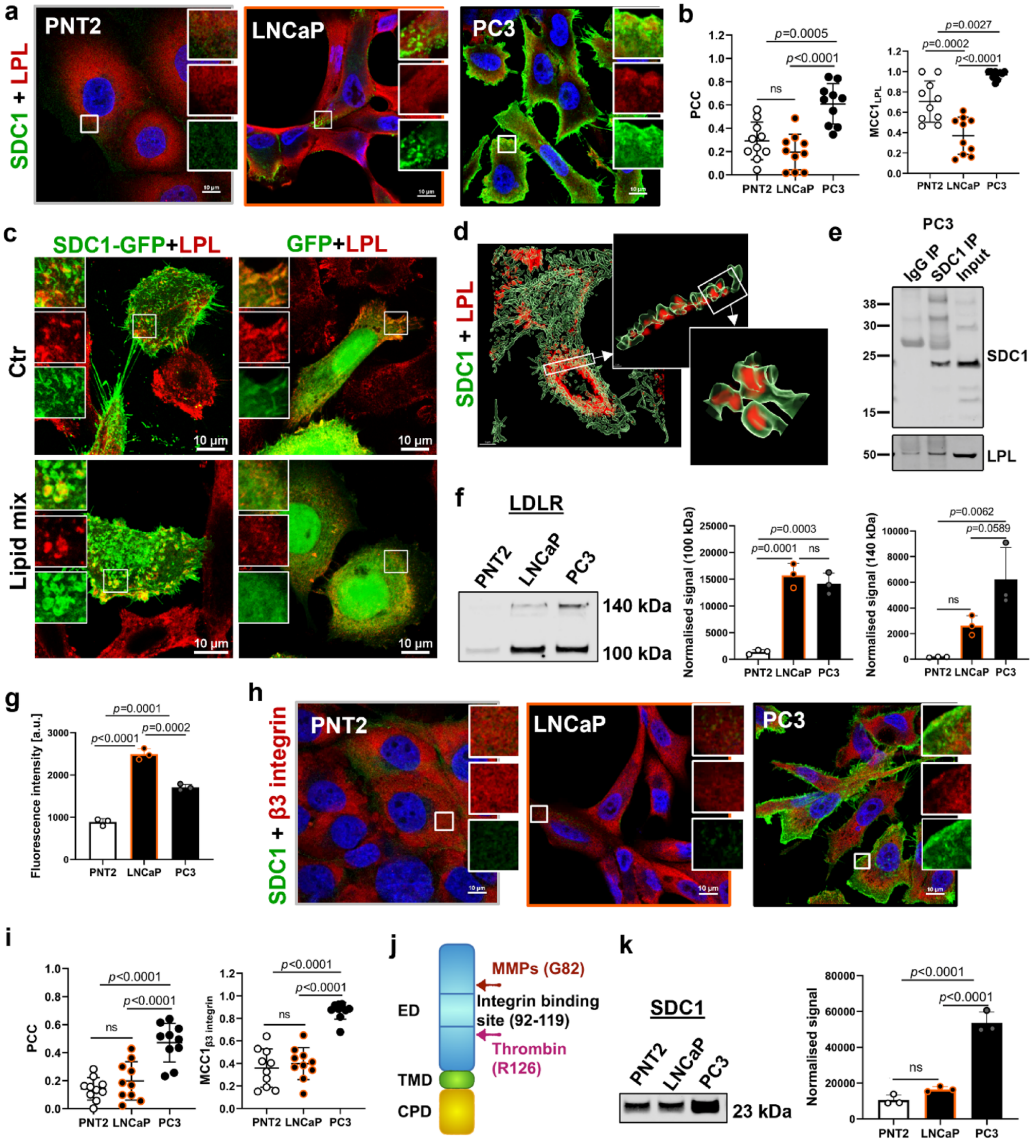
Syndecan-1 (SDC1) interacts with LPL and colocalises with integrin β_3_ in PC3 cells. (**a**) Representative confocal images showing co-labelling of cells with anti-LPL (red) and anti-SDC1 (green) antibodies. Scale bars; 10 µm. (**b**) Quantification of colocalisation between LPL and SDC1. (**c**) Confocal MaxIP images showing distribution of LPL in PC3 cells overexpressing SDC1-GFP or GFP alone, treated or untreated with lipid mix. Scale bars; 10 µm. (**d**) 3D rendering of confocal z-stacks showing SDC1 (green) encapsulating LPL (red) in PC3 cells. (**e**) Endogenous SDC1 was immunoprecipitated (IP) with anti-SDC1 antibodies and LPL was detected by Western blotting. Input; cell lysate. (**f**) Expression LDLR and a corresponding quantification of band densities. Western blotting signal was normalised using total protein staining. (**g**) Quantification of β_3_ integrin fluorescence intensity. (**h**) Representative confocal images showing co-labelling of cells with anti-β3 integrin (red) and anti-SDC1 (green) antibodies. Scale bars; 10 µm. (**i**) Quantification of colocalisation between β_3_ integrin and SDC1. (**j**) Schematic representation of SDC1 core molecule, showing integrin binding site, metalloproteinases (MMPs) and thrombin cleavage sites. (**k**) Western blotting showing a 23 kDa fragment of SDC1 and a corresponding quantification of band densities. Western blotting signal was normalised using total protein staining. Data are presented as mean ± SD, in (**b**,**i**) n = 10 representative ROIs, in (**f,g**,**k**) n = 3 (independent experiments**)**, one-way ANOVA.

There was significantly more syndecan-1 detected in PC3 cells (Figs. 1e and 5a), with stronger colocalisation between LPL and syndecan-1 in PC3 cells, compared to LNCaP and PNT2 cells (Fig. 5a,b). PC3 cells overexpressing syndecan-1-GFP and fed with lipid mix (chemically defined lipid concentrate) showed a characteristic morphology, where LPL was concentrated in vesicles surrounded by syndecan-1. This phenotype was not observed in cells over-expressing GFP alone (Fig. 5c). 3D rendering of confocal z-stacks revealed LPL vesicles surrounded by endogenous syndecan-1 in PC3 cells (Fig. 5d). As LPL can be bound to the cell membrane and considered a peripheral protein^28,29^, co-immunofluorescence on cell-surface proteins was performed, and 3D z-stack reconstruction confirmed colocalisation of LPL with syndecan-1 in small vesicular structures at the cell membrane (Supplementary Fig. S6a). Further, co-immunoprecipitation assay revealed that syndecan-1 binds to LPL at the endogenous level in PC3 cells (Fig. 5e).

Two forms of LDLR were detected in prostate cells; 100 kDa premature/truncated form and a functional 140 kDa form^30,31^ (Fig. 5f). Both LNCaP and PC3 cells expressed significantly higher amounts of a 100 kDa LDLR than PNT2 cells, while a 140 kDa form displayed significant elevation in PC3 cells as compared to PNT2 cells (Fig. 5f).

β_3_ integrins and α_v_β_3_ integrin heterodimers belong to the syndecan-1 interactome (Fig. 2a) and have an established role in PCa progression and metastasis^32^. Measurement of the total fluorescence intensity for β_3_ integrin showed significantly higher expression in malignant cells as compared to PNT2 cells, with a stronger signal in LNCaP compared to PC3 cells (Fig. 5g). There was minimal colocalisation between β_3_ integrin and syndecan-1 in PNT2 and LNCaP cells compared to PC3 cells (Fig. 5h,i).

Figure 5j illustrates a schematic syndecan-1 core molecule, where the arrow indicates a metalloproteinase (MMP2, MMP7, MMP9 and MT1-MMP) cleavage site. *MMP14* gene (encoding MT1-MMP) was highly upregulated in PC3 cells as compared to LNCaP cells (Supplementary Fig. S1 and Supplementary Table S1) and MT1-MMP was expressed only in PC3 cells; no protein was detected in PNT2 or LNCaP cells (Supplementary Fig. S6b). In PC3 cells syndecan-1 also colocalised with MT1-MMP at the cell membrane (Supplementary Fig. S6c). Cleavage by MMPs decreases the expected molecular weight of syndecan-1 to ~ 24 kDa and exposes the α_v_β_3_ integrin binding site (amino-acids 92–119)^33,34^. This fragment was recognised by our monoclonal antibody against syndecan-1 (10A3) on Western blotting and showed threefold higher band intensity for PC3 cells, as compared to PNT2 and LNCaP cells (Fig. 5k). These results agreed with significantly higher colocation between integrin β_3_ and syndecan-1 in androgen-insensitive PC3 cells (Fig. 5i).

### Androgens regulate sortilin-mediated glucose metabolism in LNCaP cells

**Figure 6:**
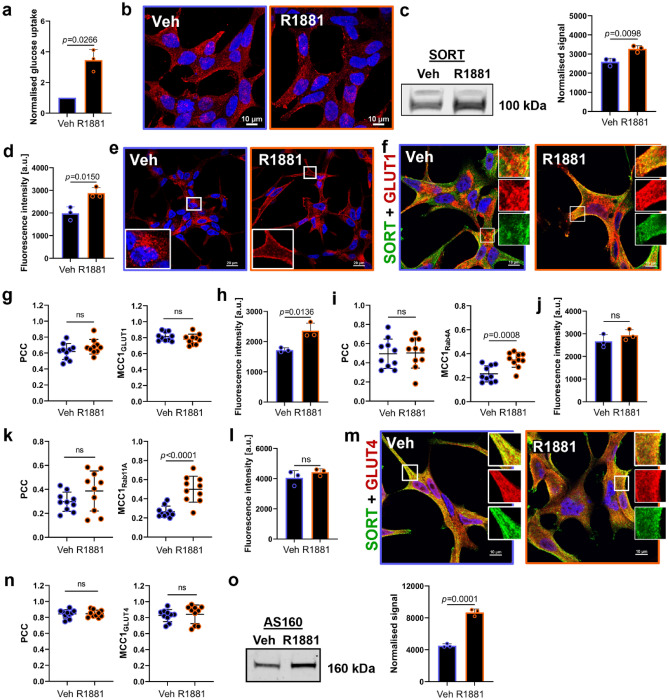
Androgens regulate sortilin (SORT)-mediated glucose metabolism in LNCaP cells. (**a**) Glucose uptake by LNCaP cells treated with vehicle (Veh) versus R1881. (**b**) Representative maxIP confocal images of LNCaP cells immunolabelled with anti-SORT antibodies. Scale bars; 10 µm. (**c**) Expression of SORT and a corresponding quantification of band densities. Western blotting signal was normalised using total protein staining. (**d**) Quantification of GLUT1 fluorescence intensity. (**e**) Representative confocal images showing distribution of GLUT1. Scale bars; 20 µm. (**f**) Representative confocal images of cells co-labelled with anti-GLUT1 (red) and anti-SORT (green) antibodies. Scale bars; 10 µm. (**g**) Quantification of colocalisation between GLUT1 and SORT. (**h**) Quantification of Rab4A fluorescence intensity. (**i**) Quantification of colocalisation between Rab4A and GLUT1. (**j**) Quantification of Rab11A fluorescence intensity. (**k**) Quantification of colocalisation between Rab11A and GLUT1. (**l**) Quantification of GLUT4 fluorescence intensity. (**m**) Representative confocal images of cells co-labelled with anti-GLUT4 (red) and anti-SORT (green) antibodies. Scale bars; 10 µm. (**n**) Quantification of colocalisation between GLUT4 and SORT. (**o**) Expression of AS160 and a corresponding quantification of band densities. Western blotting signal was normalised using total protein staining. Data are presented as mean ± SD, in (**a**,**c**,**d**,**h**,**j**,**l,o**) n = 3 (independent experiments), in (**g**,**i**,**k**,**n**) n = 10 representative ROIs, in **a** one sample t-test, all other graphs; t-test.

In PCa, the reprogramming of glucose metabolism is known to be governed by androgens^35–37^ and therefore their role in sortilin-regulated glucose metabolism was investigated. The effect of androgens on glucose uptake was confirmed by synthetic androgen (R1881) treatment, which resulted in ∽threefold higher glucose uptake, compared to untreated LNCaP cells (Fig. 6a). While R1881 treatment did not appear to alter the distribution of sortilin in LNCaP cells (Fig. 6b), Western blotting analysis of cell lysates revealed a significantly higher amount of sortilin in LNCaP cells following R1881 addition (Fig. 6c).

The fluorescence intensity of GLUT1 was significantly higher in LNCaP cells after R1881 treatment (Fig. 6d) which also caused a redistribution of GLUT1 from intracellular compartments to the cell membrane (Fig. 6e). In addition, there was high colocalisation between GLUT1 and sortilin in both R1881-treated and untreated cells; vehicle-treated LNCaP cells exhibited GLUT1 and sortilin colocalisation in the perinuclear region, while in androgen-treated cells colocalisation was also detected at the cell membrane (Fig. 6f). The amount of GLUT1 and sortilin colocalisation did not change appreciably between vehicle- and R1881-treated cells (Fig. 6g), maintaining a high overlap between GLUT1 and sortilin (MCC1_GLUT1_).

As androgens increase surface expression of GLUT1, we attempted to identify what elements of GLUT1 trafficking machinery were regulated by R1881 treatment. Rab4A and Rab11A GTPases regulate fast and slow recycling of proteins, respectively^38,39^, and R1881 treatment significantly increased total fluorescence intensity of Rab4A (Fig. 6h) and its overlap with GLUT1 (Fig. 6i). Rab11A expression was not affected by R1881-treatment (Fig. 6j), but the overlap between Rab11A and GLUT1 was ∽twofold higher in cells cultured with androgens (Fig. 6k).

The amount of GLUT4, measured by fluorescence intensity (Fig. 6l) and Western blotting (Supplementary Fig. S7a), was not affected by androgens. Confocal images of LNCaP cells showed significant colocalisation of GLUT4 and sortilin in both vehicle- and R1881-treated cells (Fig. 6m,n), but there was no difference between the treatment groups. Colocalisation between GLUT4 and Rab4A or Rab11A as well as fluorescence intensity of Rab10 and Rab14 showed no change in vehicle versus R1881-treated cells (Supplementary Fig. S7b-e). In contrast, Western blotting analysis revealed that R1881 upregulated AS160 protein by ∽twofold (Fig. 6o). RNAseq results also demonstrated that a set of genes involved in glucose pathway was upregulated upon R1881 treatment (Supplementary Fig. S8 and Supplementary Table S2).

Expression of progranulin, LPL and their colocalisation with sortilin were also tested in vehicle versus R1881-treated cells; however, no differences were observed (Supplementary Fig. S9).

### The androgen-insensitive phenotype of PCa cells involves syndecan-1 and can be altered by androgen treatment

**Figure 7:**
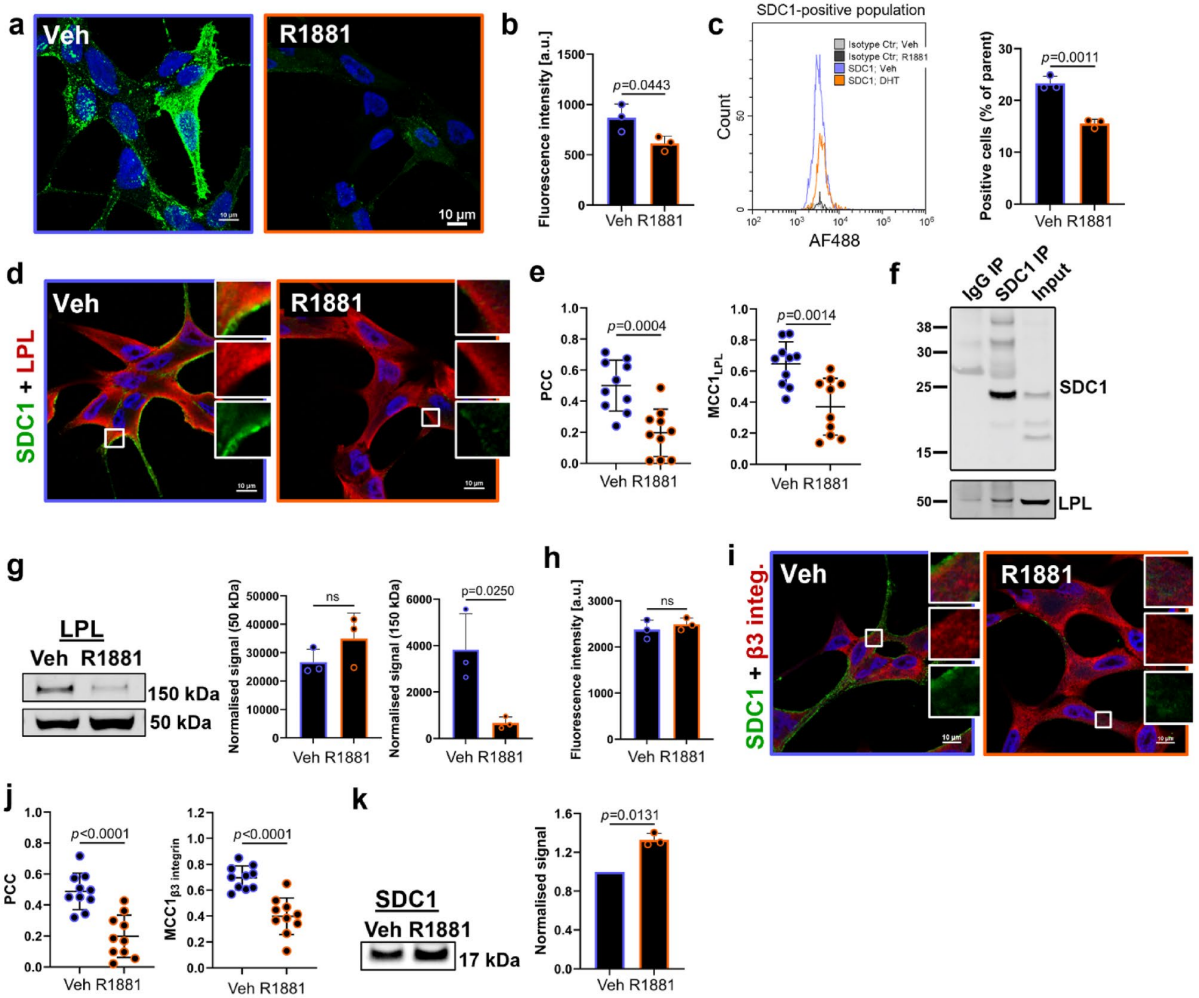
Androgen deprivation increases total and surface expression of syndecan-1 (SDC1) and its colocalisation with LPL and β3 integrin. (**a**) Representative maxIP confocal images of LNCaP cells cultured without (vehicle; Veh) or with androgens (R1881) immunolabelled with anti-SDC1 antibodies. Scale bars; 10 µm. (**b**) Quantification of SDC1 fluorescence intensity. (**c**) Representative flow cytometry histogram showing SDC1-positive cells and quantification of SDC1 positive populations. (**d**) Representative confocal images of cells co-labelled with anti-LPL (red) and anti-SDC1 (green) antibodies. Scale bars; 10 µm. **e** Quantification of colocalisation between LPL and SDC1. (**f**) Endogenous SDC1 was immunoprecipitated (IP) with anti-SDC1 antibodies and LPL was detected by Western blotting. Input; cell lysate. (**g**) Expression of LPL and a corresponding quantification of band densities. Western blotting signal was normalised using total protein staining. (**h**) Quantification of β3 integrin fluorescence intensity. (**i**) Representative confocal images of cells co-labelled with anti-β_3_ integrin (red) and anti-SDC1 (green) antibodies. Scale bars; 10 µm. (**j**) Quantification of colocalisation between β_3_ integrin and SDC1. (**k**) Western blotting showing a 17 kDa fragment of SDC1 and a corresponding quantification of band densities. Western blotting signal was normalised using total protein staining. Data are presented as mean ± SD, in (**b**,**c**,**g**,**h**,**k**) n = 3 (independent experiments), in (**e**,**j**), n = 10 representative ROIs, t-test.

Maximum intensity projection confocal images showed high total and surface expression of syndecan-1 in vehicle-treated LNCaP cells (Fig. 7a), but a marked reduction in syndecan-1 fluorescence intensity following R1881 treatment (Fig. 7a,b). *SDC1* gene was also significantly downregulated in the absence of androgens (Supplementary Fig. S8 and Supplementary Table S2). Flow cytometry measurements demonstrated significantly more cells expressing syndecan-1 in the androgen-deprived population, compared to the R1881-treated group (Fig. 7c). In addition, while the fluorescence intensity of LPL was not affected by androgens (Supplementary Fig. S9d), the colocalisation of LPL and syndecan-1 was reduced by ∽twofold, following R1881 application (Fig. 7d,e). The co-immunoprecipitation assay showed that endogenous syndecan-1 bound to LPL in LNCaP cells (Fig. 7f), even though a week signal was also detected for IgG negative control. Western blotting analysis confirmed no difference in LPL expression in vehicle versus R1881, but also showed an additional high molecular weight form in lysates from cells cultured in vehicle, which likely represents LPL dimers or oligomers^28^ (Fig. 7g).

R1881 treatment did not alter the total expression of β_3_ integrin in LNCaP cells (Fig. 7h), but reduced its colocalisation with syndecan-1 (Fig. 7i,j). As shown in Fig. 5j, processing of syndecan-1 by thrombin removes the α_v_β_3_ integrin binding site and produces a fragment with an expected molecular weight of 19 kDa. Western blotting on LNCaP cell lysates revealed a ∽17 kDa fragment of syndecan-1, likely cleaved at R126. There was significantly more of this molecular form in R1881-treated cells (Fig. 7k), indicating reduced availability of the α_v_β_3_ integrin binding site. This is in accordance with the lower colocalisation between syndecan-1 and integrin β_3_ (Fig. 7j). However, in both vehicle and R1881-treated cells the 23 kDa form of syndecan-1, with exposed integrin binding site, was more evident than the 17 kDa band (Supplementary Fig. S10); it is possible that in intact cells used for immunofluorescence, the 17 kDa form dominates at the cell surface.

## Discussion

Sortilin and syndecan-1 are indicative of the PCa cell transition from glucose to lipid metabolism and from an androgen-sensitive to an androgen-insensitive phenotype. In this study, we demonstrated that sortilin likely plays a role in glucose utilisation in PCa cells. We showed that increasing glucose concentration decreased sortilin but increased GLUT1 expression in LNCaP cells, suggesting that in androgen-responsive cells there is a glucose concentration-related link between sortilin and GLUT1 expression. We recently discovered that sortilin is overexpressed in low-grade PCa, but has a low level of expression in benign tissue and high-grade cancer^12^. Accordingly, we showed that sortilin was most abundant in LNCaP cells, when compared to PNT2 and PC3 cells; and similar expression patterns were observed for GLUT1 and GLUT4. Considering that GLUT1 is the closest homolog to GLUT4^40^, we suspected that sortilin might regulate trafficking of both of these glucose transporters in androgen-sensitive cells. Indeed, both GLUT1 and GLUT4 colocalised extensively with sortilin in LNCaP cells, including at the cell membrane, suggesting that sortilin may regulate GLUT1/4 surface expression. LNCaP cells also expressed high amounts of Rab10, Rab14 and AS160 that colocalised with sortilin, indicating that GLUT4 trafficking to the cell membrane in androgen-responsive PCa cells may occur via a similar pathway as in insulin-responsive cells^24^. This may be a result of constitutively active phosphatidylinositol-3-kinase (PI3K)/Akt signalling in PCa pathogenesis^41^ and noteworthy, LNCaP cells express the highest amounts of constitutively active Akt^42^.

Androgens increase glucose uptake^5,35,36^ and the expression of GLUT1^35,43^; and in confirming these results we demonstrated that R1881 also upregulated sortilin expression and caused re-distribution of GLUT1 from intracellular compartments to the plasma membrane, where the two proteins colocalised. We showed that elevated surface expression of GLUT1 in androgen-treated cells involved Rab11A-slow and Rab4A-fast endosome recycling. We identified AS160 as the androgen-regulated component of the GLUT4 pathway, which was significantly overexpressed in R1881-treated cells; and this is in agreement with gene^43^ and protein^36^ expression data. Considering that: (1) there was no redistribution of GLUT4 after R1881 treatment; (2) that AS160 promotes translocation of other GLUTs, including GLUT1^44^; and (3) that AS160 knockdown significantly decreased GLUT1 expression, we propose that AS160 overexpression may be more relevant for androgen-dependent GLUT1 trafficking.

There is an intricate relationship between sortilin and progranulin in PCa cells. While sortilin can bind progranulin and induce its internalisation and degradation^45^, in androgen-insensitive cells it is progranulin that can cause lysosomal degradation of sortilin. This maintains small amounts of sortilin and high levels of progranulin, which consequently promotes PCa progression^26^. Our study confirmed that sortilin interacts with progranulin and that progranulin is secreted by PC3 cells, but not by LNCaP cells. This may be a part of a molecular mechanism for down-regulating glucose metabolism and promoting lipid metabolism. The energy metabolism in castration-resistant PCa patients is heavily dependent on lipids, to drive cancer cell migration and metastasis^6^, hence high-fat diets, obesity and LPL expression are all associated with cancer progression^27,46^. Here, we showed that PCa cell lines overexpressed LPL, which interacted with sortilin, while sortilin knockdown in LNCaP cells decreased LPL. This indicates that sortilin may mediate sorting and/or internalisation of LPL^15,47^ in PCa cells. We also found that in R1881-deprived cells, LPL likely forms homodimers, which may be required for its secretion and catalytic activity^48^. Higher amounts of LPL homodimers in R1881-starved LNCaP cells implies their role in transition towards a more aggressive PCa phenotype.

Syndecan-1 sorts LPL within the Golgi apparatus into sphingomyelin-rich vesicles and supports its trafficking to the cell membrane^29^. Our study revealed that LPL and syncdecan-1 bound to each other and colocalised in PC3 cells. To the best of our knowledge, we demonstrated for the first time the association between LPL and syndecan-1 in vesicles at the cell surface of androgen-insensitive PCa cells. Syndecan-1 tethers LPL along vesicular membranes and keeps it in a filament-like inactive form^28^, thus, it is likely that in PC3 cells syndecan-1 mediates transfer of LPL to the cell surface, where it becomes active and promotes lipid uptake. We also showed that LDLR, another protein involved in lipid internalisation that interacts with syndecan-1^17^, is upregulated in PCa cell lines, and that the functional form of LDLR is expressed to the highest degree in PC3 cells. Syndecan-1 has a direct link to key proteins involved in lipid metabolism and also binds to a range of other extracellular and membrane proteins, including α_V_β_3_ integrin, that promote cancer progression^32^. Our data revealed a high colocalisation of β_3_ integrin with syndecan-1 in PC3 cells, which is in agreement with high amounts of the 23 kDa syndecan-1 form in PC3 cells, with an exposed α_V_β_3_ integrin binding site. This syndecan-1 form is probably derived from proteolytic clipping by metalloproteinases^33^. Indeed, the expression of MT1-MMP was detected only in PC3 cells, where it also colocalised with syndecan-1. Thus, this clipped form of syndecan-1 likely facilitates interactions with α_V_β_3_ integrin, which promote the invasive phenotype of androgen-insensitive cells^32^.

We demonstrated that androgen deprivation upregulated total and surface expression of syndecan-1, which is in concordance with a previous report^49^. Our study showed an association between syndecan-1 and LPL in LNCaP cells and suggested that an increased syndecan-1-mediated LPL exocytosis to the cell surface occurs in androgen-deprived cells. Syndecan-1 sorts LPL into sphingomyelin-rich vesicles^29^, while the androgen receptor can negatively regulate de novo synthesis of sphingolipids^50^, therefore, the overexpression of syndecan-1 together with upregulation of sphingolipid synthesis, likely facilitates LPL trafficking to the plasma membrane in androgen-starved cells. We also demonstrated that androgen treatment decreased the colocalisation between β_3_ integrin and syndecan-1 suggesting that androgen deprivation stimulates interactions between these two proteins, promoting an aggressive phenotype. We showed that R1881 likely stimulates syndecan-1 cleavage by thrombin which removes the α_V_β_3_ integrin binding site^34^. Hence, R1881-treated cells may have more syndecan-1 forms, which are unavailable for interaction with α_V_β_3_ integrin.

While our study provides valuable insights into the role of sortilin, syndecan-1, and associated proteins in prostate cancer cell metabolism, several limitations need to be addressed by further research, including the use of diverse cell lines, in vivo studies, and clinical validation. We recognise that whilst LNCaP and PC3 cell lines are commonly used as models for PCa research, they may not fully represent the heterogeneity and complexity of prostate cancer in patients and thus may not necessarily translate directly to clinical outcomes. This in vitro evidence presented here does not fully replicate complex tumour microenvironments or physiological conditions present in vivo. Further, our study does not address the broader aspects of genetic alterations, hormone signalling, immune responses, and microenvironment interactions. Follow-up studies are required to elucidate additional molecular mechanisms and pathways involved and to investigate if there is a direct/inverse correlation between sortilin and syndecan-1.

**Figure 8:**
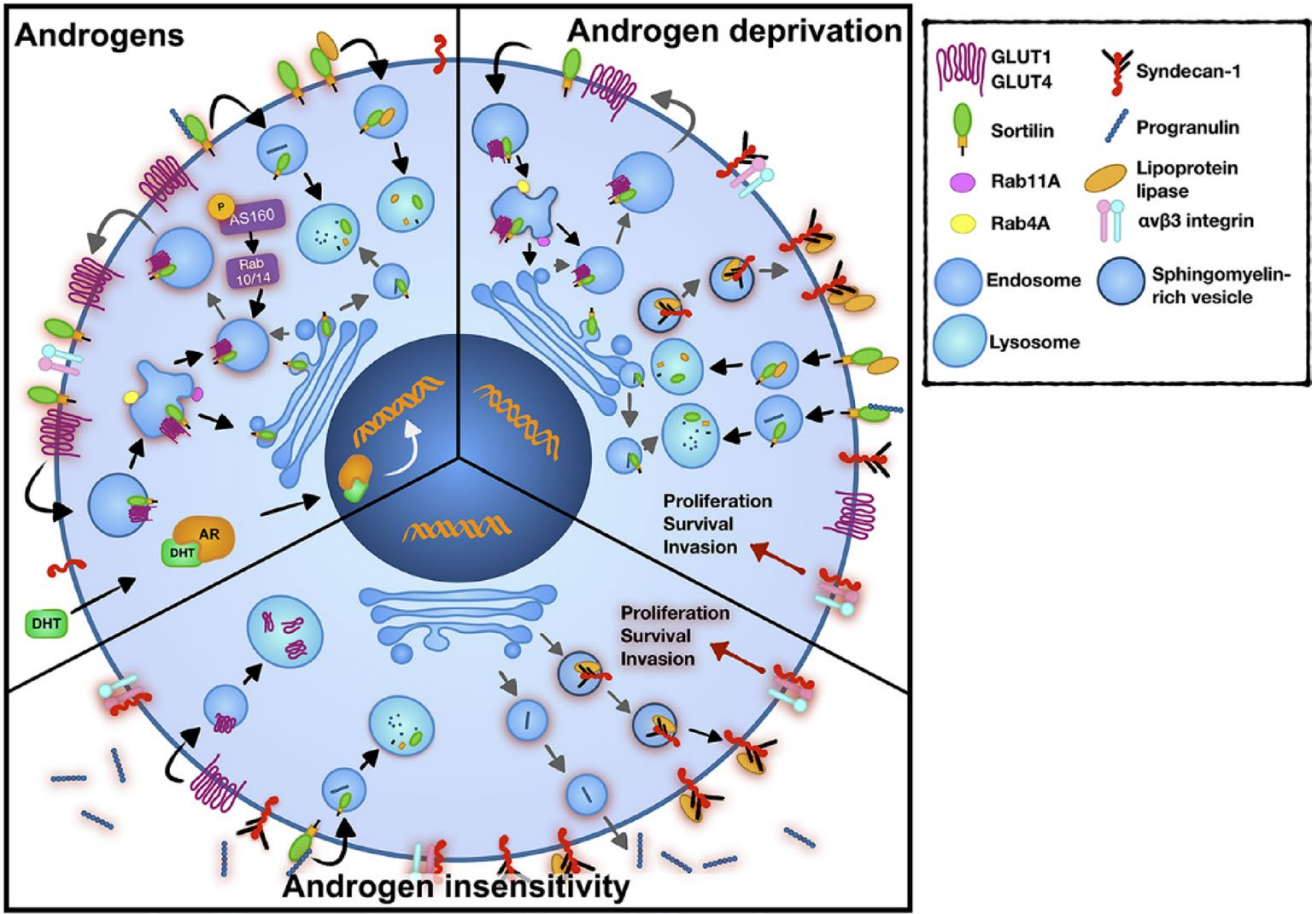
Proposed schematic summary of sortilin and syndecan-1 roles in PCa cell metabolism at different stages of progression. In androgen-sensitive cells sortilin is involved in regulation of GLUT1 and GLUT4 surface expression. Sortilin, GLUT1 and some elements of trafficking machinery are directly or indirectly upregulated by androgens (dihydrotestosterone; DHT, androgen receptor; AR). Sortilin also keeps the level of progranulin and LPL low by controlling their degradation. Androgen deprivation leads to a decrease in sortilin, GLUT1 and Rab4A expression and an increase in syndecan-1 expression. Syndecan-1 promotes trafficking of LPL to the plasma membrane, which facilitates fatty acids uptake. Syndecan-1 also interacts with α_V_β_3_ integrin, which activates proliferation, survival and invasion pathways. Androgen-insensitive PCa cells express low amounts of sortilin but high amounts of progranulin, which is released to the extracellular environment. Progranulin binds to sortilin leading to its internalisation and degradation. Aggressive PCa cells also overexpress syndecan-1, which stimulate LPL trafficking to the cell surface to enhance lipid utilisation. Cleavage of syndecan-1 by metalloproteinases exposes its α_V_β_3_ integrin binding site, which facilitates interaction between the two molecules to promote cell proliferation, survival and invasion.

In conclusion, we provide evidence for a potential role of sortilin and syndecan-1 in the metabolic programming of PCa cells that may be applicable to androgen-sensitive (or higher androgen concentration) sortilin-driven phenotypes and androgen-resistant (or lower androgen concentration) syndecan-1-reliant phenotypes. Sortilin regulates glucose metabolism and is upregulated by androgen, which also induces glucose uptake, while androgen deprivation leads to overexpression of syndecan-1 and increases its interactions with LPL and α_V_β_3_ integrin to facilitate lipid metabolism and cancer aggression (Fig. 8). This suggests that a metabolic switch may operate in PCa and this has important implications for patient management, with two metabolic phenotypes that match the two morphological patterns observed in cancer tissue; well-formed and poorly formed glands that are defined respectively by sortilin and syndecan-1. It will be important to investigate if there is a direct and inverse correlation between sortilin and syndecan-1 and to establish if there is either a molecular switch or an alternative mechanistic explanation for the alignment of these biomarkers with different stages of prostate cancer progression. Our data also has clinical implications for patients receiving or being considered for ADT, as this treatment may promote the transition to an advanced lipid metabolic phenotype, which is incurable. Therefore, both sortilin and syndecan-1 are potential companion diagnostics and novel therapeutic targets for PCa.

## Materials and methods

### Histology and immunohistochemistry (IHC)

Formalin fixed, paraffin embedded (FFPE) prostate tissue microarrays (TMA) for immunohistochemical (IHC) assessment was procured from the Flinders Medical Centre (FMC), Bedford Park, Australia. Tissue for the tissue microarray project was sourced from male patients with primary prostatic adenocarcinoma (the median age at the time of surgery was 62 years, ranging from 47 to 71 years) diagnosed between 2000 and 2005 at the Department of Anatomical Pathology, Flinders Medical Centre^51^. Approval for the construction of TMA was given by the Southern Adelaide Clinical Human Research Ethics Committee (OFR#136.16-HREC/16/SAC/). Approval specific to this study was obtained from the Human Research Ethics Committee of the University of South Australia (Application IDs: 201907 and 36070) while site specific approval was obtained from the Central Adelaide Local Health Network (Application ID R20181113; HREC/18/CALHN/749).

Serial sections (2 µm) of the TMA were cut and stained with routine haematoxylin (Ehrlich’s) and eosin (H&E) or labelled by IHC as previously described^52^. Briefly, sections underwent deparaffinisation in xylene, and rehydration through graded ethanol solutions. Heat induced epitope retrieval was carried out in Tris–EDTA Buffer (10 mM Tris Base, 1 mM EDTA Solution, 0.05% Tween®-20, pH 9.0) in a Sharp model R-9270 microwave oven heated on high for 4 min and medium–low for a further 15 min. Tissue sections underwent endogenous peroxidase blocking with 3% H_2_O_2_ for 5 min before incubation with 200 µl of primary antibody for 1 h at room temperature (RT) (sortillin; 0.273 µg/ml, syndecan-1; 2.958 µg/ml)^12^. Immunolabelling detection was performed using the DAKO EnVision + and Liquid diaminobenzidine (DAB) + System kits (Dako Australia Pty Ltd., NSW, Australia) as per manufacturer’s instructions. Tissue sections were counterstained with Ehrlich’s haematoxylin, rinsed in water and dehydrated through a graded series of ethanol solutions. Sections were cleared in xylene and a coverslip applied over DPX mounting media (Merck Millipore Pty Ltd., VIC, Australia). All slides were imaged in brightfield with a ZEISS Axio Scan Z.1 slide scanner with a Plan-Achromat 20 × objective (Zeiss, Jena, Germany). H&E sections and IHC sections with sortilin and syndecan-1 labelling were assessed by a histopathologist, and representative regions were annotated for benign glands, Gleason pattern 3, Gleason pattern 4, and Gleason pattern 5 glands.

### Cell cultures

Human prostate cell PNT2 (ECACC 95012613), LNCaP (clone FCG, ECACC 89110211) and PC3 (ECACC 90112714) were obtained from the European Collection of Authenticated Cell Cultures (ECACC) from CellBank Australia (Children's Medical Research Institute, Westmead, NSW, Australia). All cell lines were authenticated by short tandem repeat (STR) profiling (ECACC) and were negative for mycoplasma contamination (tested at 4–6 month intervals with a Lonza mycoplasma kit). PNT2 and LNCaP cells were maintained in RPMI 1640 medium (Gibco), while PC3 cell line was maintained in Ham’s F12K medium (Gibco); media were supplemented with 10% fetal bovine serum (FBS, Moregate Biotech). Media were replaced every 2 to 3 days and cells were sub-cultured at 70–80% confluency. Cell cultures were maintained at 37 °C with 5% CO_2_. For androgen treatment of LNCaP cells, 24 h after seeding RPMI medium was replaced with RPMI containing no phenol red and supplemented with charcoal-stripped FBS (CS-FBS, Gibco). After another 24 h, 10 nM synthetic dihydrotestosterone (R1881, Sigma) or corresponding concentration of vehicle (0.01% ethanol) was added. Cells were incubated with vehicle or R1881 for 48 h. At the same time, for PNT2 and PC3 cell lines media were replaced with regular RMPI or Ham’s F12K, respectively, to ensure fresh nutrient supply. For all experiments cells were seeded at densities, so that at the end of incubation time (day 5) each cell line reached ~ 80% confluency: for polystyrene flasks/plates the seeding densities were: PNT2; 0.56 × 10^4^ cells/cm^2^, LNCaP; 2.8 × 10^4^ cells /cm^2^, PC3; 1.3 × 10^4^ cells/cm^2^.

### siRNA and plasmids

SMARTpool ON-TARGETplus siRNA against Sortilin (6272, L-010620-00-0005), AS160 (9882, L-021230-01-0005) and non-targeting Pool (DHA-D-001810-10-05) were purchased from DharmaCon Inc. Sortilin-GFP, GLUT4-mCherry, Syndecan-1-GFP and GFP pcDNA3.1 plasmids were constructed by GeneArt; the fluorescence tag was attached to the C-terminus of the protein. pBABE-puro_HA-GLUT1 and pBABEpuro_HA-GLUT4 plasmids were a gift from Professor David James (The University of Sydney). HA-GLUT1 and HA-GLUT4 sequences were cloned from pBABE-puro plasmids into pcDNA3.1+. Briefly, pBABE-puro_HA-GLUT1 was digested with AfeI and PsiI and HA-GLUT1 fragment was cloned into pcDNA3 EcoRV, treated with calf intestinal alkaline phosphatase. pBABEpuro_HA-GLUT4 was digested with BamHI and SalI and HA-GLUT4 fragment was cloned into pcDNA3 BamHI and XhoI. Sequencing verified integrity and orientation of the cDNA’s.

### Cell transfection

For knockdown experiments a reverse transfection was performed. 25 nM of scrambled (control) siRNA, siRNA against Sortilin (siSORT) or AS160 (siAS160) were diluted in Opti-Mem medium (Gibco) and then mixed with Lipofectamine RNAiMAX Transfection Reagent (Invitrogen) first diluted in Opti-Mem medium (Gibco). The mixture was incubated for 40 min. at RT and then added to LNCaP cells freshly seeded onto 6-well plates (6.72 × 10^4^ cells/well). Cells were incubated for 72 h after addition of transfection mix. For overexpression experiments, LNCaP cells (1.4 × 10^4^ cells/well) were seeded 20 h before transfection on CellView 10-well, glass-bottom slides (Greiner). Plasmids (0.5 µg/cm^2^ for single transfection or 0.3 µg/cm^2^ of each plasmid for co-transfection) and Lipofectamine 2000 Transfection Reagent (Invitrogen) were diluted in Opti-Mem medium (Gibco) in separate tubes, then mixed together and incubated for 20 min at RT. The mixture was added to cells and after 5 h incubation, fresh medium containing 10% FBS was added to cells. For cells transfected with syndecan-1-GFP or GFP media containing 2% chemically defined lipid concentrate (Gibco) (final concentration 1%) or fresh media were added to cells 24 h after transfection and the cells were incubated for additional 20 h before the assay.

### Glucose uptake assay

To determine glucose uptake Glucose Uptake-Glo Assay (Promega) was used. LNCaP cells transfected with siRNA, as described above, were seeded onto CellStar 96-well glass-bottom, white plate (Greiner) at 2 × 10^4^ cells/well in RPMI media containing no glucose and supplemented with 10% FBS. Cells were incubated for 20 h before the assay. To compare glucose uptake in LNCaP cells treated with vehicle versus R1881, cells were seeded onto 6-well plates and treated with vehicle or R1881 as described in the “Cell cultures” section. On the day of the glucose assay LNCaP cells were trypsinised, centrifuged (125 g, 5 min), resuspended in glucose-free RPMI and 5 × 10^4^ cells/well were seeded onto a 96-well glass-bottom, white plate (Greiner) We measured glucose uptake in cells using the Glucose Uptake-Glo™ Assay (Promega, J1341) according to the manufacturer’s instructions. Briefly, cells were incubated with or without treatment for the indicated time and then exposed to 2-deoxyglucose (2DG) for 15 min at 37 °C. The uptake of 2DG was stopped by adding Stop Buffer, which also lysed the cells and destroyed any NADPH. The acid was neutralised by adding Neutralisation Buffer before adding the 2DG6P Detection Reagent, which contained glucose-6-phosphate dehydrogenase (G6PDH), NADP+, Reductase, Ultra-Glo™ Recombinant Luciferase and proluciferin substrate. G6PDH oxidised 2-deoxyglucose-6-phosphate (2DG6P) to 6-phosphodeoxygluconate and simultaneously reduced NADP + to NADPH. The Reductase used NADPH to convert the proluciferin to luciferin, which was then used by Ultra-Glo™ Recombinant Luciferase to produce a luminescent signal that was proportional to the concentration of 2DG6P. Incubation time with the detection reagent was 1 h. Luminescence was measured at 560 nm at 1 s. integration using Victor3 (PerkinElmer) plate reader and normalised to signal recorded for cells treated with vehicle (set arbitrary to 1).

### Immunofluorescence

For immunofluorescence, cells were seeded on glass-bottom 96-well plates (Greiner). Cells were fixed for 12 min with ice-cold solution containing 8% paraformaldehyde (PFA, Electron Microscopy Sciences) and 8% sucrose (Sigma) in PBS, which was slowly added to cells in warm media at 1:1 ratio. After fixation, cells were washed 3 × 5 min in PBS, blocked and permeabilised for 1 h in 2.5% bovine serum albumin (BSA, Sigma) in PBS containing 0.05% saponin (Sigma) (blocking buffer). Cells were incubated with primary antibodies diluted in blocking buffer overnight at 4 °C, then washed 3 × 5 min in PBS, followed by 1 h incubation with secondary antibodies diluted in blocking buffer, in the dark, at RT. Next, cells’ nuclei were stained for 10 min with Hoechst 33342 (Invitrogen) dye, washed 3 × 5 min in PBS and once in milli-Q water before adding 40 µl of Fluoromount-G (Merck) to each well. For co-immunofluorescence experiments, single-labelled samples were prepared and imaged separately to control for crosstalk between fluorophores. Samples treated with only secondary antibodies were used as a negative control. A list of primary and secondary antibodies used for immunofluorescence is provided in Supplementary Table S3.

### Surface immunofluorescence

For surface immunofluorescence cells were seeded as for immunofluorescence and on the day of experiment incubated with primary antibodies in full media at 4 °C for 1 h. Next, cells were washed 3 times in PBS, fixed in solution containing 4% PFA and 4% sucrose in PBS for 8 min, and washed again 3 × 5 min in PBS. Then, cells were blocked in 2.5% BSA in PBS for 1 h, at RT and incubated for a further 1 h with secondary antibodies The remainder of the procedure was the same as described in the “Immunofluorescence” section. Anti-sortilin antibody labelling with Alexa Fluor 488 fluorophore was performed using Alexa Fluor 488 antibody labelling kit (A20181, Life Technologies) according to manufacturers’ instructions.

### Cell discoverer 7 (CD7) imaging

For determination of fluorescence intensity and colocalisation between proteins, samples were captured with CD 7 (Zeiss) high throughput automated microscope using auto-corrective 20 × Plan-Apochromat Objective (NA 0.95). LED light intensity and exposure time were adjusted for each fluorophore so that the fluorescence was in the dynamic range. Definite focus with software auto-focus strategy were used to verify positions on the 96-well plate (ensuring that fields of view were in focus), which were then captured automatically with the same settings for all samples.

### Quantification of fluorescence intensity

The fluorescence intensity for each image captured with CD7 was quantified with Zen Blue software (ver. 3.0, Zeiss) using automatic analysis module, which applies the same settings to all captured images; a minimum intensity threshold was set based on negative controls to cut off background fluorescence. The fluorescence intensity signal for each cell line (~ 80% confluency) is an average of five images per one experiment, which was repeated 3 times independently.

### Quantification of colocalisation

Pearson correlation coefficient (PCC), which reports on the proportional co-distribution of the two fluorophores, and Mander’s colocalisation coefficients (MCC), which measures the fraction of one fluorophore that colocalises with the second fluorophore, independently of signal proportionality^53^, were determined using Zen Blue software (ver. 3.0, Zeiss) equipped with colocalisation tool. To remove background signal, in each experiment the minimum intensity threshold for each fluorophore was set based on a single-labelled control and applied to the software colocalisation window. Colocalisation was quantified for 10 representative regions of interest (ROIs) for each tested condition. As the green signal almost fully colocalised with red signal, we only reported the fraction of red-labelled protein that colocalised with green-labelled protein (MCC1).

### Confocal imaging

For detailed visualisation of cellular structures cells were imaged with a Nikon A + confocal microscope (Nikon, Japan), equipped with a LU-N4/LU-N4S 4-laser unit (403, 488, 561 and 638 nm), the A1-DUG GaAsP Multi Detector Unit (2 GaAsP PMTs + 2 standard PMTs), a 32 channel spectral detector (Nikon, Minato, Tokyo, Japan) and a Uno‐Combined‐Controller, CO_2_ microscope electric top stage incubation system (Okolab, Pozzuoli, NA, Italy) to maintain live cells at 37 °C with 5% CO_2_. Images were acquired using Plan Apo λ 60 × oil immersion objective, with 2 × line averaging. Z-stacks were captured with 0.2 µm step and 2 × line averaging. The video showing GLUT4-mCherry with sortilin-GFP was acquired for 5 min with no delay, 2 × line averaging and 3 × zoom. Images were processed using NIS Elements software (ver. 4.5, Nikon). 3D rendering of z-stack images was performed using Imaris software (ver. 9.7.0, Oxford Instruments).

### Flow cytometry

For flow cytometry, LNCaP cells were cultured in T25 flasks and treated with R1881 or vehicle as described above. On the day of assay, cells were detached and resuspended in cold FACS buffer (PBS, 1% BSA and 0.1% sodium azide), followed by fixation with 4% PFA. After washing, cells were resuspended in FACS buffer containing 0.05% saponin and 20 µg/ml of anti-syndecan-1 antibody (ab34164) or isotype control antibody (ab170190) and were incubated for 30 min at RT. Next, cells were washed, resuspended in FACS buffer containing 0.05% saponin and incubated with 1 µg/ml donkey anti-mouse Alexa Fluor 488 secondary antibody (Invitrogen, A-21202) for 30 min at RT in the dark. After washing, cells were resuspended in FACS buffer and 10.000 events per sample were acquired using Cytoflex S flow cytometer (Beckman Coulter). Data were analysed using CytExpert 2.4 (Beckman Coulter). An example of the gating strategy for this experiment is presented in Supplementary Fig. S11.

### Cell treatment with increasing glucose concentration

Cells were seeded in T25 flasks. After 48 h of incubation media were changed to RPMI media (Gibco) containing no glucose or supplemented with a desired concentration of glucose (5.5 mM, 11.1 mM or 22.2 mM). Cells were incubated in different glucose concentration for 48 h before collection of cell lysates.

### Collection of cell lysates and conditioned media

For the collection of lysates on the day of assay, cells were washed with ice cold PBS before addition of RIPA lysis buffer (Milipore) supplemented with Halt™ protease and phosphatase inhibitor cocktail (Thermo Scientific). Cell lysates were centrifuged at 10,000×*g* for 10 min, at 4 °C and the supernatant was transferred to fresh Eppendorf tubes and stored at − 30 °C. For the collection of conditioned media and corresponding lysates, cells were cultured in T75 flasks. 48 h before collection, media were replaced with serum-free RPMI media containing no phenol red (Gibco). On the day of assay, conditioned media were harvested into falcon tubes, centrifuged at 2000 g, for 10 min at 4 °C and then filtered through 0.22 µm syringe filters to remove cell debris. Next, media were concentrated using Vivaspin Turbo 15 concentrators (Sartorius) with a 3 kDa cut-off by centrifugation at 4000 g at 4 °C, until ~ 1.4 ml of concentrate was achieved. Halt™ protease and phosphatase inhibitor cocktail and 5 mM EDTA (Thermo Scientific) were added the concentrated conditioned media, which were then stored at − 30 °C. The protein concentration in cell lysates and conditioned media was determined using Micro BCA Protein Assay Kit (Thermo Scientific) as per manufacturer’s instructions. Collected lysates and conditioned media were blotted for progranulin and lipoprotein lipase.

### SDS-PAGE and Western blotting

Equal amount of protein (10—20 µg, depending on the protein of interest) was loaded onto 8% or 10% Bolt™ Tris-Bis Mini Protein Gel (Invitrogen) and the electrophoresis was run at 130 V, 1 h. Next, the protein was transferred onto PVDF membrane using iBlot dry blotting system (Life Technologies). Electrophoresis for proteins in conditioned media and corresponding cell lysates was run simultaneously and the gels were placed on the same membrane for protein transfer. This was followed by total protein staining with Revert total protein stain (Li-Cor Biosciences) and signal recording with Odyssey CLx System (Li-Cor Biosciences) as per manufacturer’s instructions.

After de-staining the membrane was blocked in an appropriate blocking buffer (5% BSA or 5% skimmed milk in TBS buffer), for 1 h at RT, then rinsed with TBS buffer containing 0.1% Tween (TBS-T), followed by overnight incubation at 4 °C with primary antibodies (Supplementary Table S3), diluted in blocking buffer. Next, the membrane was washed 3 × 5 min in TBS-T and then incubated for 1 h with secondary antibodies (Supplementary Table S3) in the dark, at RT. This was followed by washing 3 × 5 min in TBS-T and once in deionized water. Imaging was performed using Odyssey CLx System (Li-Cor Biosciences) at 84 µm resolution. Image processing and quantification of band densities was performed using Empiria Studio (ver. 1.3, Li-COR Biosciences). Western blotting signal was normalised using total protein staining. Western blot conditions for each protein are provided in Supplementary Table S4.

### Co-immunoprecipitation

Cells were cultured in T175 flasks and at 80% confluency cells were washed once with cold DPBS and then incubated with 2 mM of dithiobis(succinimidyl propionate (Pierce™ Premium Grade DSP, Thermo Fisher Scientific) for 1 h at 4 °C. Then, quenching buffer (1 M Tris, pH 7.5) was added for 15 min at 4 °C. Cells were lysed in 1.2 mL of Pierce ™ IP Lysis Buffer (Thermo Fisher Scientific) containing 1% (v/v) Halt™ protease and phosphatase inhibitor cocktail (Thermo Fisher) followed by one wash with cold DPBS. Cell lysates were centrifuged and stored as described above. For the immunoprecipitation 500 µg of protein was incubated with 2–4 µg of primary or nonspecific IgG isotype control antibodies (Supplementary Table S3) overnight at 4 °C on HulaMixer™ Sample Mixer with rotating angle at 45° and speed at 25 rpm (Thermo Fisher). Lysate-antibody complex was then incubated with 100 µL of μMACS Protein G MicroBeads (Miltenyi Biotec) for 1 h at 4 °C using HulaMixer™ Sample Mixer at 25 rpm. Postabsorptive supernatant was magnetically isolated after eluting the lysate-antibody-bead complex through µMACS™ separator and µ column (Miltenyi Biotec) according to the manufacturer's instructions. Epitope location and specificity of the in-house antibodies against sortilin and syndecan-1 used in this method are provided in Supplementary Figs. S12 and S13.

### Statistics

Statistical analysis was performed using GraphPad Prism 9 software (version 9.3.1) Data are presented as mean of at least three independent experiments ± SD. For fluorescence intensity quantification, for each cell line signal from five images was averaged per one independent experiment. For colocalisation analysis data are presented as mean of 10 representative ROIs. For comparison between three cell lines the statistical significance was assessed using one-way ANOVA with Tukey’s multiple comparisons (for comparison of every mean with every other mean) or Dunnett’s multiple comparisons (for comparison of every mean to a control mean) test, where p ≤ 0.05 was considered significant. For comparison between two conditions (LNCaP cells treated with R1881 versus vehicle), t-test or one-samples t-test (for normalised data) was performed, where differences were deemed significant at *p* ≤ 0.05. Outliers were determined based on Dixon’s Q test (https://contchart.com/outliers.aspx).

### Supplementary Information

Supplementary Information 1.

Supplementary Information 2.

Supplementary Video 1.

## Data Availability

The RNA sequencing data cell lines generated in this study have been deposited in the Gene Expression Omnibus (GEO) – NCBI – NIH repository under the accession number GSE220618. All experimental data that support the findings of this study are included as follows: 7 main figures, 13 supplementary figures and 4 supplementary tables. The Source data file is provided with this paper.
