# Supplementary material for: Dynamic interplay between sortilin and syndecan-1 contributes to prostate cancer progression
Source: Sci Rep. 2023 Aug 18;13:13489. doi: 10.1038/s41598-023-40347-7 (PMC10439187; doi:10.1038/s41598-023-40347-7)
Supplement: Supplementary file 2 — Supplementary Information 2. [file 41598_2023_40347_MOESM2_ESM.pdf]

Supplementary information for:

Dynamic interplay between sortilin and syndecan-1 contributes to prostate cancer progression

Joanna Lazniewska[1*], Ka Lok Li[1], Ian R. D. Johnson[1], Alexandra Sorvina[1], Jessica M. Logan[1], Carmela Martini[1], Courtney Moore[1], Ben S-Y. Ung[1], Shane M. Hickey[1], Litsa Karageorgos[1], Sarita Prabhakaran[1,2], Jessica K. Heatlie[1], Robert D. Brooks[1], Chelsea Huzzell[1], Nicholas I. Warnock[3], Mark P. Ward[4], Bashir Mohammed[4], Prerna Tewari[4], Cara Martin[4], Sharon O ' T ó[4], Laure Bogue Edgerton[4], Mark Bates[4], Paul Moretti[3], Stuart M. Pitson[3], Stavros Selemidis[5], Lisa M. Butler[6,7], John J. O ' L é[4], Douglas A. Brooks[1*]

[1]Clinical and Health Sciences, University of South Australia, Adelaide SA 5000, Australia. [2]Department of Anatomical Pathology, College of Medicine and Public Health, Flinders University, Bedford Park SA 5042, Australia. [3]Centre for Cancer Biology, University of South Australia and SA Pathology, Adelaide SA 5000, Australia. [4]Department of Histopathology, Trinity College Dublin, Dublin 8, Ireland. [5]School of Health and Biomedical Sciences, STEM College, RMIT University, Bundoora VIC 3083, Australia. [6]South Australian ImmunoGENomics Cancer Institute and Freemasons Centre for Male Health and Wellbeing, University of Adelaide, Adelaide SA 5000, Australia. [7]Solid Tumour Program, Precision Cancer Medicine theme, South Australian Health and Medical Research Institute, Adelaide SA 5000, Australia. *e-mail: joanna.lazniewska@unisa.edu.au, doug.brooks@unisa.edu.au

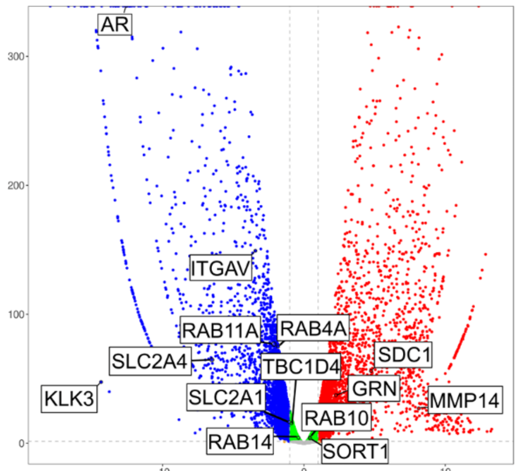

Supplementary Figure S1. Differential expression of genes in prostate cancer cell lines. Volcano plot showing differentially expressed genes in PC3 versus LNCaP cells. Genes of interests are annotated.

Supplementary Table S1. Differential expression of genes of interest in PC3 versus LNCaP cells.

Gene	Log FC	LogCPM	*p* value	FDR
KLK3	-14.100	11.020	1.10E-66	7.88E-66
AR	-12.494	7.027	Infinity	Infinity
SLC2A4	-6.363	2.978	1.48E-95	1.73E-94
ITGAV	-3.431	8.299	8.85E-168	2.63E-166
RAB11A	-2.075	7.944	8.50E-85	8.23E-84
RAB4A	-1.832	6.913	2.17E-77	1.87E-76
SLC2A1	-1.036	6.108	4.15E-18	1.05E-17
TBC1D4	-0.869	6.705	6.00E-17	1.47E-16
RAB14	-0.376	7.466	3.58E-05	5.33E-05
LDLR	0.052	7.457	7.65E-01	7.82E-01
RAB10	0.413	8.105	1.13E-05	1.72E-05
SORT1	0.439	5.654	9.91E-05	1.44E-04
GRN	2.262	8.084	4.57E-42	2.04E-41
SDC1	4.748	8.957	5.46E-63	3.69E-62
MMP14	7.929	5.541	1.19E-39	5.10E-39

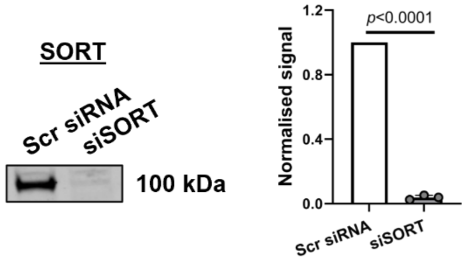

Supplementary Figure S2. Western blotting showing succesful knockdown of sortilin in LNCaP cells. Scr siRNA; scrambled siRNA, siSORT; sortilin siRNA. Sortilin was detected using Abcam ab16640 antibody. Total protein staining was used for Western blot signal normalisation. Data are presented as mean ± SD, n = 3, one-sample t-test.

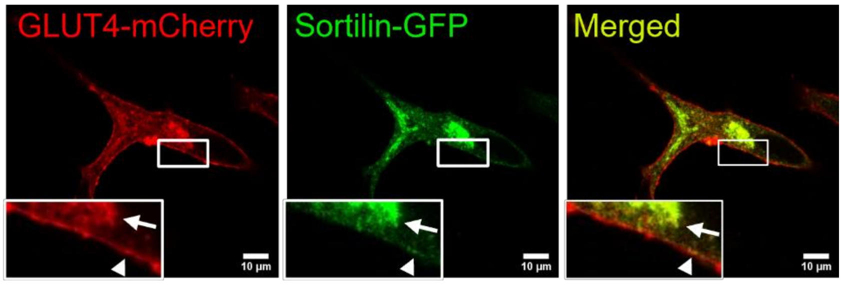

Supplementary Figure S3. GLUT4-mCherry (red) colocalises with sortilin-GFP (green) in intracellular stores (arrows) and at the plasma membrane (arrowheads) in LNCaP cells. Scale bar; 10 µm.

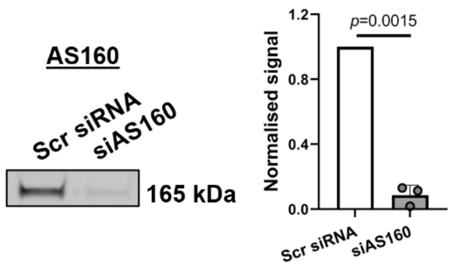

Supplementary Figure S4. Western blotting showing succesful knockdown of AS160 in LNCaP cells. Scr siRNA; scrambled siRNA, siAS160; AS160 siRNA. Total protein staining was used for Western blot signal normalisation. Data are presented as mean ± SD, n = 3, one-sample t-test.

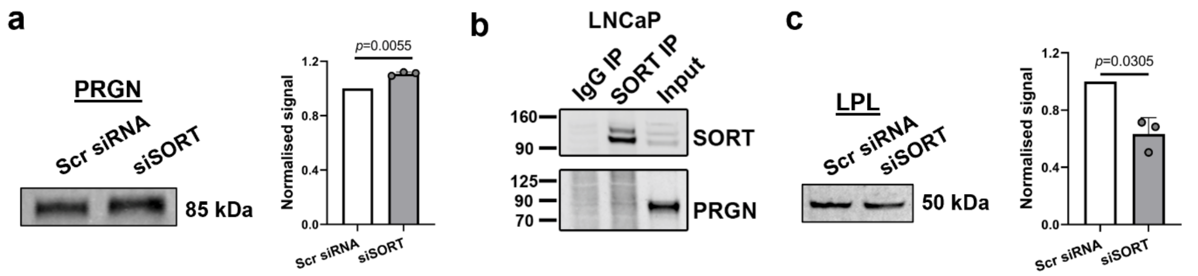

Supplementary Figure S5. Sortilin (SORT) knockdown affects progranulin (PRGN) and lipoprotein lipase (LPL) expression, but SORT does not bind to PRGN in LNCaP cells. **a** SORT knockdown in LNCaP cells leads to a higher level of progranulin (PRGN). **b** Association between endogenous SORT and PRGN was not detected in LNCaP cells. SORT was immunoprecipitated (IP) with monoclonal anti-SORT antibodies and PRGN was detected by Western blotting. Cell lysates (Input) were also subjected to Western blotting. **c** SORT knockdown in LNCaP cells leads to decreased level of LPL. Scr siRNA; scrambled siRNA, siSORT; sortilin siRNA. Total protein staining was used for Western blot signal normalisation. Data are presented as mean ± SD, n = 3, one-sample t-test.

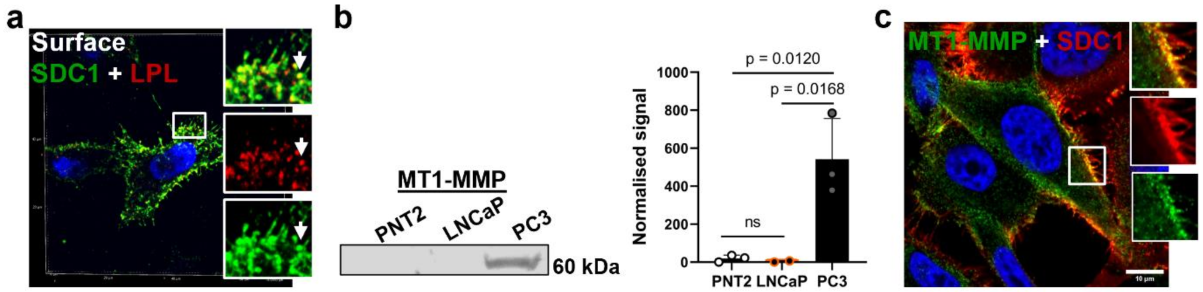

Supplementary Figure S6. Syndecan-1 (SDC1) colocalises with lipoprotein lipase (LPL) and is likely processed by MT1-MMP in PC3 cells. a Confocal 3D reconstruction from z-stack showing colocalisation between SDC1 (green) and LPL (red) in vesicular structures (example; white arrows) at the cell surface of PC3 cells. **b** Western blotting showing the expression of MT1-MMP in prostate cell lines and a corresponding quantification of band densities. Western blotting signal was normalised using total protein staining. **c** A representative confocal image showing PC3 cells co-labelled with anti-SDC1 (red) and anti-MT1-MMMP (green) antibodies. Scale bar; 10 µm. Data are presented as mean ± SD, n = 3 (independent experiments**)**, one-way ANOVA.

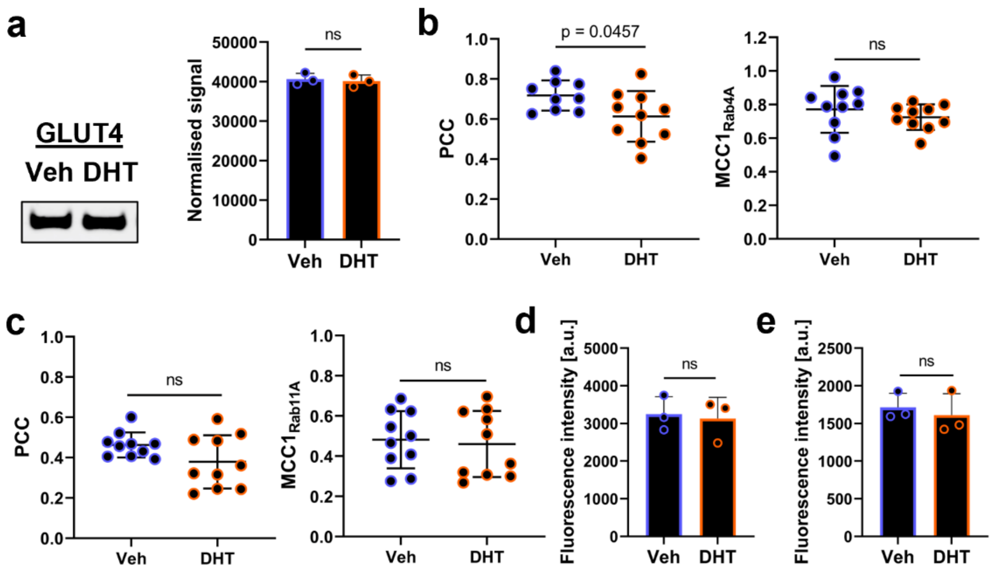

Supplementary Figure S7. Androgens do not alter total expression and recycling machinery of GLUT4. a Western blotting showing total expression of GLUT4 in LNCaP cells cultured with vehicle (Veh) or DHT. **b** Quantification of colocalisation between Rab4A and GLUT4 in Veh versus DHT. **c** Quantification of colocalisation between Rab11A and GLUT4 in Veh versus DHT. **d** Fluorescence intensity of Rab10 in Veh versus DHT. **e** Fluorescence intensity of Rab14 in Veh versus DHT. Data are presented as mean ± SD, in **a**, **d** and **e** n = 3 (independent experiments), in **b** and **c** n = 10 (10 ROIs from two independent experiments), t-test.

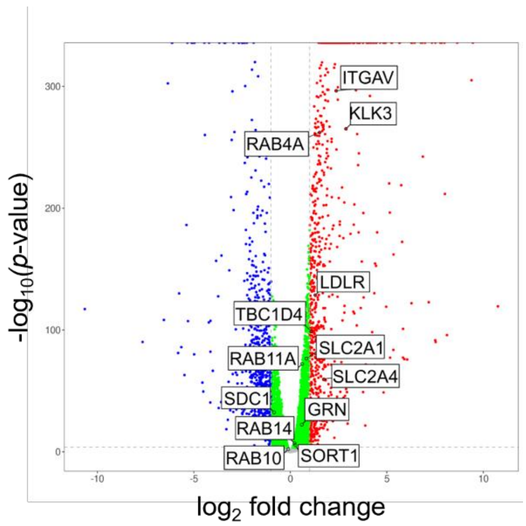

Supplementary Figure S8. Volcano plot showing differential expression of genes in LNCaP cells treated with DHT versus vehicle. Genes of interests are annotated.

Supplementary Table S2 Differential expression of genes of interest in DHT-treated versus vehicle-treated LNCaP cells.

Gene	LogFC	LogCPM	*p* value	FDR
SDC1	-0.842	5.386	2.26E-33	1.14E-32
RAB10	-0.106	7.908	5.34E-03	7.53E-03
RAB14	0.215	7.577	7.45E-08	1.51E-07
SORT1	0.249	5.356	2.23E-06	4.12E-06
GRN	0.593	6.402	3.09E-23	1.19E-22
RAB11A	0.609	8.487	1.32E-77	1.59E-76
SLC2A1	0.828	6.377	1.22E-75	1.44E-74
TBC1D4	1.082	6.862	1.14E-97	1.81E-96
LDLR	1.287	7.197	7.05E-133	1.60E-131
RAB4A	1.514	7.290	4.62E-258	2.47E-256
SLC2A4	1.732	3.723	3.33E-54	2.63E-53
ITGAV	2.375	8.800	1.76E-303	1.22E-301
KLK3	2.893	11.642	1.05E-274	6.09E-273

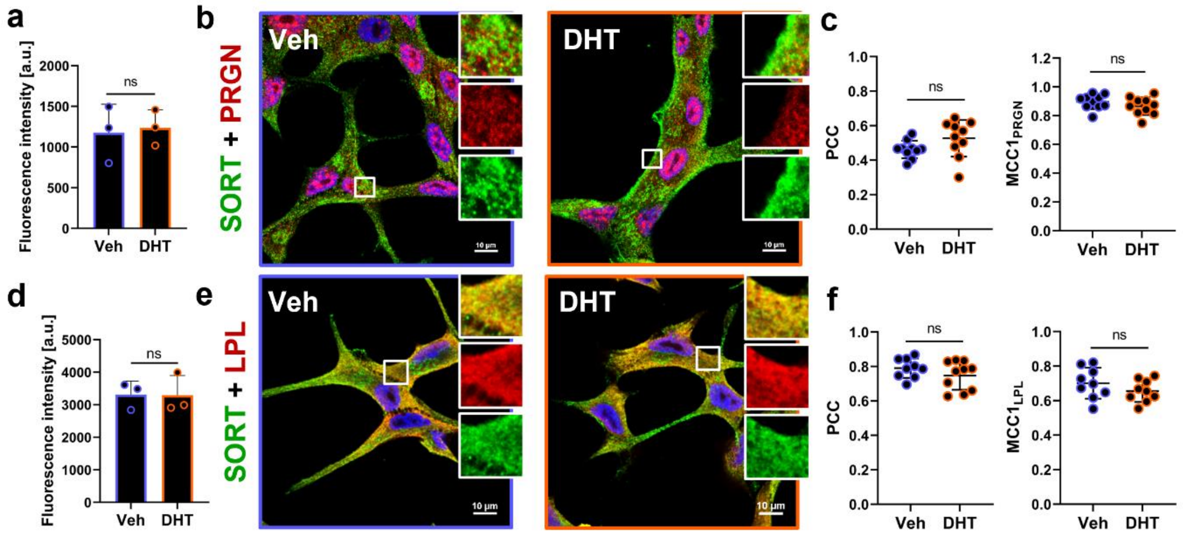

Supplementary Figure S9. Progranulin (PRGN) and lipoprotein lipase (LPL) expression and their colocalisation with sortilin (SORT) are not androgen-dependent. a Fluorescence intensity of PRGN in vehicle (Veh) versus DHT. **b** Representative confocal images showing LNCaP cells in Veh versus DHT co-labelled with anti-PRGN (red) and anti-SORT (green) antibodies. Scale bars; 10 µm. **c** Quantification of colocalisation between PRGN and SORT in Veh versus DHT. **d** Fluorescence intensity of LPL in Veh versus DHT. **e** Representative confocal images showing LNCaP cells in Veh versus DHT co-labelled with anti-LPL (red) and anti-SORT (green) antibodies. Scale bars; 10 µm. **f** Quantification of colocalisation between LPL and SORT in Veh versus DHT. Data are presented as mean ± SD, in **a** and **d** n = 3 (independent experiments), in **c** and **f** n = 10 (10 ROIs from two independent experiments), t-test.

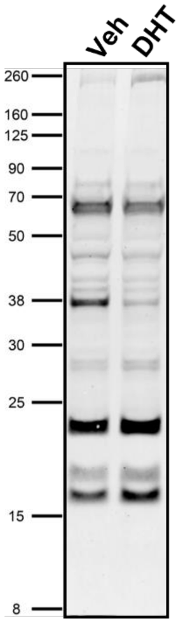

Supplementary Figure S10. Western blotting showing expression of syndecan-1, detected by 10A3 antibodies in LNCaP cells treated with vehicle (Veh) versus DHT.

Supplementary Table S3. Antibodies used in the study.

**Primary Antibodies**		
Antibody	Source	Application and concentration
Mouse monoclonal anti-sortilin 9E10	In-house-designed[1]	Immunofluorescence: 2 μg/ml<br>IHC: 0.273 μg/ml<br>Immunofluorescence: 2 μg/ml
Mouse monoclonal anti-sortilin 9E10 Alexa Fluor488	In-house-designed[1]	Surface immunofluorescence: 10 μg/ml
Mouse monoclonal anti-sortilin 8C3, 9E10, 4B12, 11H8	In-house-designed[1]	Immunoprecipitation: 2 μg/ml of each antibody
Mouse IgG1 monoclonal [MOPC-21]	Abcam, ab18443	Immunoprecipitation: 2-4 μg/ml
Mouse IgG2a monoclonal [MOPC-173]	Abcam, ab18413	Immunoprecipitation: 2 μg/ml
Mouse IgG2b monoclonal [MG2b-67]	Abcam, ab18421	Immunoprecipitation: 2-4 μg/ml
Rabbit polyclonal anti-sortilin	Abcam, ab16640	Western blot: 0.5 μg/ml
Rabbit polyclonal anti-HA tag	Abcam, ab9110	Surface immunofluorescence: 10 μg/ml
Mouse monoclonal anti-syndecan-1 [B-A38]	Abcam, ab34164	Immunofluorescence: 0.4 μg/ml<br>Flow cytometry: 20 μg/ml
Mouse IgG1 monoclonal [15-6E10A7]	Abcam, ab170190	Flow cytometry: 20 μg/ml
Mouse monoclonal anti-syndecan-1 10A3-2	In-house-designed[1]	IHC: 2.958 μg/ml<br>Immunofluorescence: 2 μg/ml<br>Western blot: 1 μg/ml
Mouse monoclonal anti-syndecan-1 5G4, 6D11, 10A3	In-house-designed[1]	Immunoprecipitation: 2 μg/ml of each antibody
Rabbit monoclonal anti-GLUT1 [EPR3915]	Abcam, ab115730	Immunofluorescence: 0.395 μg/ml<br>Western blot: 0.0158 μg/ml
Mouse monoclonal anti-GLUT1 [SPM498]	Abcam, ab40084	Immunofluorescence: 2.5 μg/ml
Rabbit polyclonal anti-GLUT4	Abcam, ab33780	Immunofluorescence: 2 μg/ml
Mouse monoclonal anti-GLUT4 [1F8]	Abcam, ab35826	Immunofluorescence: 4 μg/ml
Rabbit polyclonal anti-Rab10	Invitrogen PA5-102855	Immunofluorescence: 4 μg/ml
Rabbit polyclonal anti-Rab14	Abcam, ab28639	Immunofluorescence: 2 μg/ml
Rabbit polyclonal antibody anti-AS160	Invitrogen, A303-728A	Immunofluorescence: 1 μg/ml<br>Western blot: 0.5 μg/ml
Rabbit polyclonal antibody anti-Rab4A	Invitrogen PA5-28863	Immunofluorescence: 4 μg/ml
Rabbit polyclonal antibody anti-Rab11A	Abcam, ab180778	Immunofluorescence: 2 μg/ml
Rabbit monoclonal anti-granulin [EPR15864]	Abcam, ab208777	Immunofluorescence: 0.43 μg/ml
Mouse monoclonal anti-granulin [2D4-2F1]	Invitrogen, MA1-187	Immunofluorescence: 50 μg/ml<br>Western blot: 1 μg/ml
Rabbit monoclonal anti-lipoprotein lipase [JA22-02]	Invitrogen, MA5-32688	Immunofluorescence: 10 μg/ml<br>Western blot: 1 μg/ml
Rabbit polyclonal anti-LDLR	Abcam, ab30532	Western blot: 0.4 μg/ml
Rabbit polyclonal antibody anti-integrin β 3	Invitrogen, PA5-79539	Immunofluorescence: 1 μg/ml
Rabbit monoclonal anti-MMP14 [EP1264Y]	Abcam, ab51074	Western blot: 0.08 μg/ml<br>Immunofluorescence: 0.2 μg/ml
**Secondary antibodies**		
Donkey anti-mouse Alexa Fluor 488	Invitrogen, A-21202	Immunofluorescence: 1 μg/ml<br>Flow cytometry: 1 μg/ml
Donkey anti-mouse Alexa Fluor 647	Invitrogen, A-31571	Immunofluorescence: 2 μg/ml
Donkey anti-rabbit Alexa Fluor 488	Invitrogen, A-21206	Immunofluorescence: 2 μg/ml
Donkey anti-rabbit Alexa Fluor 647	Invitrogen, A-31573	Immunofluorescence: 2 μg/ml
IRDye 680RD goat anti-rabbit IgG	Li-cor, 926-68071	Western blot: 0.1 μg/ml
IRDye 800CW goat anti-mouse IgG2b	Li-cor, 926-32352	Western blot: 0.1 μg/ml
IRDye 800CW goat anti-mouse IgG1	Li-cor, 926-32350	Western blot: 0.1 μg/ml

Supplementary Table S4. Western blot conditions for proteins used in the study.

Protein detected	95 °C, 5 min	Bis-Tris Gel [%]	Protein/well [µg]	Blocking buffer (in Tris-buffered saline)
Sortilin	Yes	10	15	5% BSA
Syndecan-1	Yes	10	15	5% BSA
GLUT1	No	10	15	5% BSA
GLUT4	No	10	10	5% milk
AS160	Yes	8	10	5% milk
Progranulin	Yes	10	10	5% BSA
Lipoprotein lipase	Yes	10	10	5% BSA
LDLR	Yes	8	10	5% BSA
MT1-MMP	Yes	10	20	5% BSA

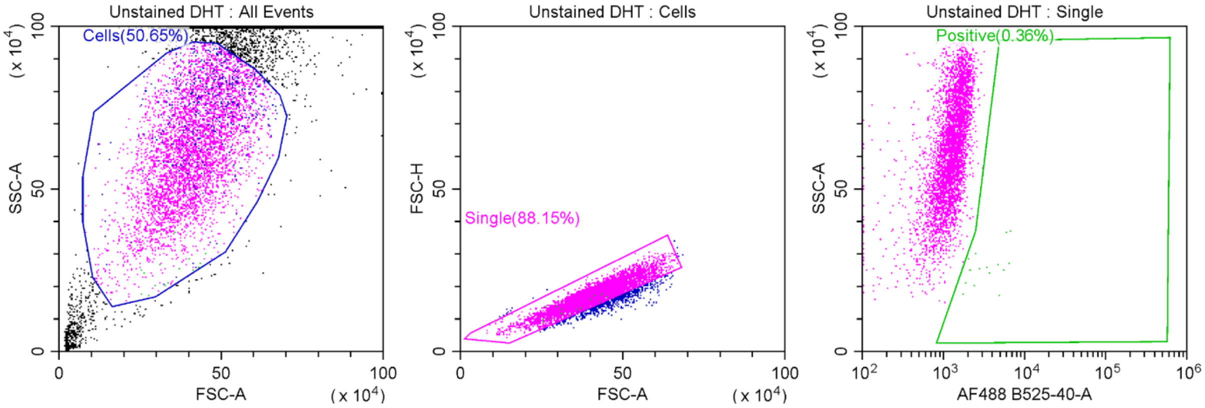

Supplementary Figure S11. An example of the gating strategy used for flow cytometry experiments

a **b**

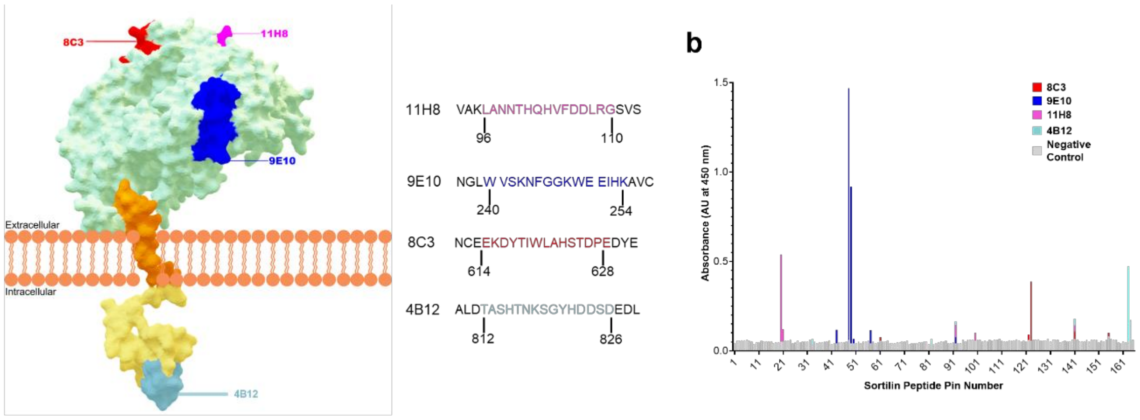

c

Clone	Sortilin peptide pin number recognised by antibody (peptide sequence)	Absorbance (AU at 450 nm)	
		Antibody	Control
11H8	Pin Number 20 (LANNTHQHVFDDLRG)	0.538	0.051
9E10	Pin Number 48 (ENGLWVSKNFGGKWE)	1.468	0.059
9E10	Pin Number 49 (VSKNFGGKWEEIHKA)	0.918	0.042
8C3	Pin Number 123 (NCEEKDYTIWLAHST)	0.386	0.057
4B12	Pin Number 163 (DTASHTNKSGYHDDS)	0.472	0.054

Supplementary Figure S12. Design and specificity of anti-sortilin monoclonal antibodies. **a** Model of sortilin created using PyMOL structure prediction. Monoclonal antibodies were raised against specifically selected linear peptide sequences - SORT-11H8 (pink), SORT-9E10 (blue), SORT-8C3 (red), and SORT-4B12 (cyan). **b** Epitope specificity and reactivity of the monoclonal anti-sortilin antibodies: 11H8 (pink), 9E10 (blue), 8C3 (red) and 4B12 (cyan), expressed as ELISA absorbance at 450 nm and shown for individual peptide pins. **c** Peptide sequences found to interact with the monoclonal antibodies 11H8, 9E10, 8C3 and 4B12 based on ELISA.

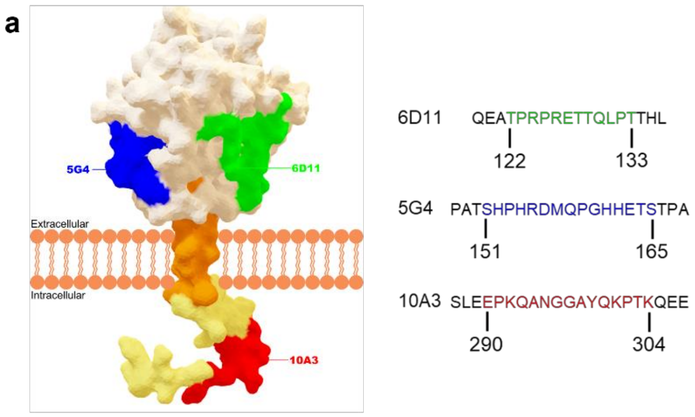

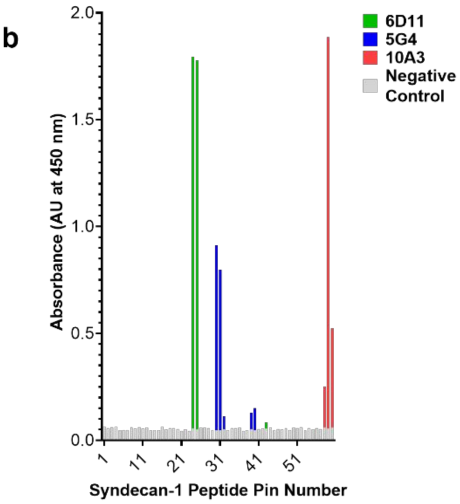

c

Clone	Syndecan-1 peptide pin number recognised by antibody (peptide sequence)	Absorbance (AU at 450 nm)	
		Antibody	Control
6D11	Pin Number 24 (AREQEATPRPRETTQ)	1.793	0.054
6D11	Pin Number 25 (ATPRPRETTQLPTTH)	1.777	0.051
5G4	Pin Number 30 (QEPATSHPHRDMQPG)	0.912	0.049
5G4	Pin Number 31 (SHPHRDMQPGHHETS)	0.797	0.047
10A3	Pin Number 59 (PKQANGGAYQKPTKQ)	1.886	0.055
10A3	Pin Number 60 (GGAYQKPTKQEEFYA)	0.523	0.06

Supplementary Figure S13. Design and specificity of anti-syndecan-1 monoclonal antibodies. **a** Model of syndecan-1 created using Phyre^2 structure prediction. Monoclonal antibodies were raised against specifically selected linear peptide sequences - 6D11 (green), 5G4 (blue) and 10A3 (red) **b** Epitope specificity and reactivity of the monoclonal anti-syndecan1-1 antibodies: 6D11 (green), 5G4 (blue) and 10A3 (red), expressed as ELISA absorbance at 450 nm and shown for individual peptide pins. **c** Peptide sequences found to interact with the monoclonal antibodies 6D11, 5G4, and 10A3 based on ELISA.

Supplementary methods

Design and production of sortilin and syndecan-1 monoclonal antibodies

The specific epitopes on sortilin and syndecan-1 were selected with the aid of AbDesigner and Phyre^2 to model entire protein structures and to define linear sequence for antibody production. The production of all monoclonal antibodies was outsourced to GenScript for GLP standard production (Piscataway, NJ, USA) and developed in C57BL/6 mice[1,2].

RNA sequencing (RNAseq)

For RNAseq cells were seeded into 6-well plates and RNA was collected using PureLink RNA Mini Kit (Thermo Fisher Scientific). RNA quality control and RNA sequencing was outsourced to the Australian Cancer Research Foundation (ACRF) Cancer Genomics Facility (South Australia). Sequencing was performed on an Illumina Nextseq sequencer, producing an average of 28 million 74 nucleotide single-end reads per sample (range 21 –33 million reads). Raw reads were analysed using FastQC (version v0.11.9) to assess quality and adapter contamination. Adapters were trimmed using cutadapt (version 1.18)[3] with the following parameters: -a AGATCGGAAGAGCACACGTCTGAACTCCAGTCA --minimum-length 18 --error-rate 0.2 --overlap 5. Trimmed reads were aligned to the hg38 reference genome using STAR (version 2.7.10a)[4]. PCR duplicates were removed using UMI-tools (version 1.1.2)[5], before deduplicated reads were re-mapped using STAR, producing a gene counts matrix. Differential gene expression analysis of these gene counts was performed in R using the Bioconductor package edgeR (version 3.38.4)[6]. Lowly expressed genes were first filtered by requiring greater than 3 counts per million in at least three libraries. Library size normalisation factors were calculated using the *calcNormFactors* function, enabling accurate comparisons of gene expression between samples. Data were explored using a multidimensional scaling (MDS) plot generated using *plotMDS*, allowing assessment of replicate sample clustering and treatment effects. A negative binomial generalised log-linear model was fit to the read counts for each gene using *glmFit*, utilising a design matrix including a term describing experimental batch. A likelihood ratio test was performed using *glmLRT* to test the difference in gene expression between treatments. Genes with an adjusted p-value (FDR) < 0.05 and fold-change of at least 2 between treatments were designated as differentially expressed.

References

1. Martini, C. *et al.* Aberrant protein expression of Appl1, Sortilin and Syndecan-1 during the biological progression of prostate cancer. *Pathology* (2022) doi:10.1016/j.pathol.2022.08.001.

2. Johnson, I. R. D. *et al.* A paradigm in immunochemistry, revealed by monoclonal antibodies to spatially distinct epitopes on syntenin-1. *Int. J. Mol. Sci.* **20**, 6035 (2019).

3. Martin, M. Cutadapt Removes Adapter Sequences From High-Throughput Sequencing Reads. *EMBnet.journal* **17**, 10–12 (2011).

4. Dobin, A. *et al.* STAR: Ultrafast universal RNA-seq aligner. *Bioinformatics* **29**, 15–21 (2013).

5. Smith, T., Heger, A. & Sudbery, I. UMI-tools: Modeling sequencing errors in Unique Molecular Identifiers to improve quantification accuracy. *Genome Res.* **27**, 491–499 (2017).

6. Robinson, M. D., McCarthy, D. J. & Smyth, G. K. edgeR: A Bioconductor package for differential expression analysis of digital gene expression data. *Bioinformatics* **26**, 139–140 (2009).

7. Armstrong, H. K. *et al.* Dysregulated fibronectin trafficking by Hsp90 inhibition restricts prostate cancer cell invasion. *Sci. Rep.* **8**, 1–14 (2018).
